# Machine Learning-Guided Design of Rhenium Tricarbonyl Complexes for Next-Generation Antibiotics

**DOI:** 10.1021/acsbiomedchemau.5c00125

**Published:** 2025-09-04

**Authors:** Miroslava Nedyalkova, Gozde Demirci, Youri Cortat, Kevin Schindler, Fatlinda Rahmani, Justine Horner, Mahdi Vasighi, Aurelien Crochet, Aleksandar Pavic, Olimpia Mamula, Fabio Zobi, Marco Lattuada

**Affiliations:** 1 Department of Chemistry, Fribourg University, Chemin Du Musée 9, Fribourg 1700, Switzerland; 2 Swiss National Center for Competence in Research (NCCR) Bio-inspired Materials, 27211 University of Fribourg, Fribourg 1700, Switzerland; 3 Department of Computer Science and Information Technology, 113403 Institute for Advanced Studies in Basic Sciences (IASBS), Zanjan 45137-66731, Iran; 4 Institute of Molecular Genetics and Genetic Engineering, University of Belgrade, Vojvode Stepe 444a, Belgrade 11042, Serbia; 5 Haute Ecole d’Inginerie et d’Architecture Fribourg (HEIA), Institute of Chemical Technology, University of Applied Sciences Western Switzerland, Fribourg CH-1700, Switzerland

**Keywords:** rhenium complexes, metal complex, antimicrobial, machine learning, antimicrobial resistance

## Abstract

The escalating prevalence of antibiotic-resistant bacteria and the increasing complexity of managing severe infections emphasize the critical need for novel and effective antibiotics. Herein, we present a novel computational strategy focused on metal-based antibiotics, specifically rhenium (Re) complexes, for the rational design of next-generation antibacterial agents. Our approach integrates machine learning (ML) classification models to predict antibacterial potency, particularly against multidrug-resistant pathogens. A recognized limitation of conventional ML-driven antibiotic discovery is its dependence on structural similarity to known antibiotics, which hinders the exploration of structurally diverse and innovative antibiotic classes. To address this, we developed predictive ML models based on multi-layer perceptron (MLP) and random forest (RF) algorithms to estimate the minimum inhibitory concentration (MIC) of Re complexes against methicillin-resistant (MRSA) and methicillin-sensitive (MSSA) *Staphylococcus aureus* strains. Utilizing structural descriptors, these models demonstrated strong predictive performance and were successfully applied to evaluate 26 novel Re complexes. Additionally, Shapley additive explanation (SHAP) analysis provided insights into the structural features influencing antibacterial activity predictions. The study’s outcomes affirm the effectiveness of our ML-guided approach as a promising pathway for the rational, *de novo* design of potent Re based antibiotics capable of combating antibiotic-resistant bacterial infections.

## Graphical abstract

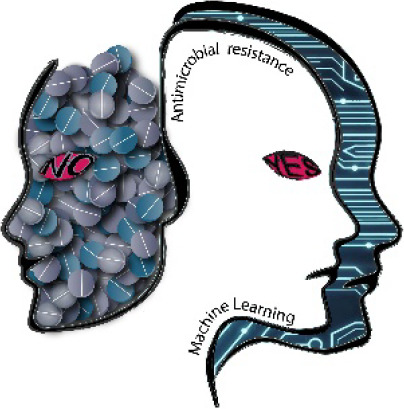

The emergence of antibiotic-resistant pathogens is widely recognized as a significant global threat, necessitating the development of novel strategies for creating new classes of antibiotics. Managing infections caused by resistant pathogens, such as carbapenem-resistant *Enterobacteriaceae* (CRE), methicillin-resistant *Staphylococcus aureus* (MRSA), multidrug-resistant tuberculosis (MDR-TB), vancomycin-resistant *Enterococcus* (VRE), extended-spectrum beta-lactamase (ESBL)-producing bacteria, and drug-resistant species including *Candida auris*, *Neisseria gonorrheae*, *Plasmodium falciparum*, and *Toxoplasma gondii*, is increasingly challenging. The UN’s 2023 report, Bracing for Superbugs: Strengthening Environmental Action in the One Health Response to Antimicrobial Resistance, forecasts a dramatic rise in deaths from drug-resistant infections by 2050, potentially resulting in a global economic loss of $3.4 trillion and pushing an additional 24 million people into extreme poverty unless decisive actions are implemented. In 2019 alone, approximately 1.27 million deaths globally were directly attributed to drug-resistant infections, with 4.95 million deaths linked specifically to bacterial antimicrobial resistance (AMR). For perspective, in the same year, deaths related to HIV/AIDS and malaria totaled approximately 860,000 and 640,000, respectively, according to the World Health Organization (WHO). Resistance mechanisms primarily include genetic mutations, horizontal gene transfer, and inappropriate antibiotic usage, further exacerbating the emergence and spread of multidrug-resistant ‘superbugs.^1,2^

Despite the urgent need for novel antibiotics and advanced diagnostic tools to rapidly detect infections, only 45 traditional antibiotics were undergoing clinical development as of 2021.

Therefore, alternative approaches, such as ML applications to the discovery of anti-infective drugs, have centered on training models to identify potential new drugs or new uses for existing drugs. Since the number of drug-like small molecules is virtually infinite, 10 ^60^ and possibly greater,^1^ most antibiotics are not considered drug-like,^2^ because they do not conform to standard drug-likeness rules, such as Lipinski’s Rule of Five, which defines properties associated with orally active small-molecule drugs. Given the growing need to develop ML models trained on nontraditional scaffolds and physicochemical properties tailored explicitly for antibiotic activity. This could include incorporating data on metal-containing compounds, natural product-like frameworks, or molecules with unusual functional groups that exhibit antimicrobial effects. Notably, the development of the first computer-designed antibiotic with proven efficacy in preclinical animal models underscores the potential of ML in guiding the discovery of novel therapeutic molecules.^3^

Stokes et al. demonstrated the potential of deep learning for antibacterial compound prediction by training a neural network on *Escherichia coli* growth inhibition data from 2335 unique compounds, including FDA-approved drugs and natural products.^4^ Their model identified halicin as an effective antibacterial agent, with validated activity both *in vitro* and *in vivo*. More recently, Capecchi et al. used ML to predict nonhemolytic antimicrobial peptides, further showcasing its versatility in identifying novel therapeutic candidates.^5^

Inorganic or organometallic metal complexes play a pivotal role as potential alternatives for new antimicrobial agents. However, metals and metalloantibiotics have only recently gained considerable attention as potential antimicrobials in response to the rapid rise of AMR in the past decade. Organometallic compounds hold promise thanks to the flexibility of their chemistry, which allows one to change their structure and the nature of their ligands.^6,7^ Among such species, rhenium-based complexes^8–14^ hold great potential. Their mechanism of action is not fully understood, but current evidence points to the bacterial membranes as the target of compounds.^15–17^ One of the first detailed mechanism-of-action investigations for metalloantibiotics was undertaken by the groups of Bandow and Metzler-Nolte.^18^ They revealed that a trimetallic complex containing rhenium, iron, and manganese, with a peptide nucleic acid backbone, showed good activity against a range of Gram-positive bacteria, including MRSA, vancomycin-intermediate *S. aureus*, and *Bacillus subtilis*. Unfortunately, the reported results about the activity against the tested Gram-negative pathogens did not show the same measured response. In addition to in-depth mechanistic studies, a structure–activity relationship was carried out, demonstrating that the Recontaining [(dpa)­Re­(CO)_3_] moiety was crucial for the activity responses, while ferrocenyl and CpMn­(CO)_3_ units could be replaced by nonmetal moieties such as a phenyl ring.^8^ The application of ML for predicting bioactive metal complexes was recently published for the case of ruthenium.^19^

The discovery of new antimicrobial agents is a pivotal necessity, and the potential of metal-based complexes as drugs, particularly as antibiotics, should be explored. We should address the challenges outlined earlier to boost development in the field. It is critically important to prescribe appropriate antimicrobial therapy as quickly as possible. Whole-genome sequencing approaches for rapidly identifying pathogens and predicting antibiotic resistance phenotypes are becoming increasingly feasible. They may effectively reduce turnaround times for clinical tests relative to traditional culture-based methods, thereby improving patient outcomes. Using whole genome sequencing data from 1668 clinical isolates of *Klebsiella pneumoniae*, Nguyen et al. developed a machine learning model based on XGBoost that accurately predicted antibiotic MICs.^20^ The obtained MICs predicted by the model correlate with recognized antimicrobial resistance genes. Pataki et al. used 704 *E. coli* genomes combined with measured MIC for ciprofloxacin collected from different countries for a MIC prediction model based on Random Forest.^21^ The model was developed to identify the genomic features that determine disease susceptibility. The recent progress in whole genome sequencing technology in combination with machine learning analysis approaches indicates that soon, such an approach might become cheaper and faster than a MIC measurement.^22–24^

As we have seen, in recent years, ML could deliver an alternative approach to streamline the development process of *de novo* antibiotics by identifying the key motif in the molecular structure associated with antibiotic activity. The application of ML to drug discovery, specifically antibiotic discovery, has been greatly facilitated by the public availability of empirical data sets,^25–28^ such as the Open Antimicrobial Drug Discovery (CO-ADD) for metal-containing compounds with antimicrobial activity.^6,29^ Antibacterial screening approaches still lack efficient tools and strategies for rapidly identifying new chemotypes. Implementing the ML methods for novel compounds acting against Gram-negative bacteria is scarcely used.^30,31^ An ML-guided approach based on descriptor space search and selection has already been used to predict antimicrobial activity.^32–35^ These findings underscore not only the promise of metal-containing scaffolds as a new frontier in antibiotic discovery but also the importance of ML in systematically unraveling the complex structure–activity relationships that govern noncovalent interactions in such systems.^36^

To our knowledge, this is the first ML study tailored to Re tricarbonyl complexes as antibacterial agents, a scaffold with distinct coordination chemistry and pharmacological behavior relative to previously modeled metal systems. The cornerstone of this study is to explore the descriptor space as a tool for systematically representing the chemical and structural diversity of rhenium tricarbonyl complexes in a format suitable for machine learning. Given the nonstandard geometry, electronic properties, and coordination behavior of metal-based compounds, classical drug-likeness rules and fingerprint-based representations are often insufficient. To address this, we generated an extensive set of descriptors—spanning topological indices, physicochemical properties, electronic parameters, and spatial pharmacophoric features—derived from optimized geometries and saved as.mol files, which served as input for molecular descriptor calculation using AlvaDesc. Based on the diversity of the descriptors space and top line results for the descriptors ranking as aromaticity (e.g., BLI), molecular branching (e.g., ChiA indices), charge distribution (e.g., MATS and ATS series), and surface area contributions (e.g., VSA-related descriptors), which are hypothesized to correlate with the antimicrobial efficacy, particularly membrane interaction and permeability, is the main line of the novelty described here.

To interpret the impact of these descriptors on model predictions, we implemented the Shapley technique that assigns an importance value to each feature for individual predictions. The correlation with SHAP analysis suggests that active complexes were predominantly influenced by descriptors related to aromaticity, polarizability, and molecular flexibility—such as BLI, MATS 3p, and SM1_Dz—indicating their critical role in bacterial membrane disruption. Conversely, nonactive complexes were associated with descriptors indicating high rigidity, poor charge dispersion, or suboptimal pharmacophoric alignment (e.g., Eig05_AEA (ed), CIC3).

This study not only finds the application of ML to Retricarbonyl complexes for MIC-level antimicrobial prediction but also introduces descriptors closely linked to the activity, dual-strain modeling (MSSA and MRSA), and a computational-experimental feedback loop that shows a methodological approach and a conceptually essential way in metal-based antibiotic discovery design. The whole concept was developed and can be used in a low-data regime.

For which the adjustment of learning methods was adapted with proper architectures through descriptor dimensionality reduction, facilitating advanced predictive performance in new complex systems.

Our results affirm the effectiveness of this ML-guided approach as a powerful strategy for the rational, *de novo* design of potent, structurally novel Re based antibiotics capable of combating antibiotic-resistant bacterial infections.

## Materials and Methods

### Data Set Structure

The literature was searched for a large representative number of complexes of the *fac*-[Re­(CO)_3_]^+^ core evaluated *in vitro* for their anticancer cytotoxic properties (IC_50_ values). Data were extracted from various sources, primarily from available reviews on the subject.^37–39^ The work of Wilder et al. also provided several data points for our analysis.^40^ Redicarbonyl complexes of the *cis*-[Re­(CO)_2_]^+^ core were also added to the search.^41,42^ Both mononuclear (ca. 95% of the final total) and dinuclear species, mainly with mono- and bidentate ligands, were included in the pool. This initial selection of candidate molecules was further analyzed in terms of the cancer cell lines used in their *in vitro* evaluation. The cytotoxicity of molecules is known to be cell line-dependent; therefore, we evaluated all the data and identified the four most common cell lines for which the largest number of data points is available. These are the HeLa, MCF7, MCF10A, and MDA-MB-231cancer cell lines. A final total of 228 cases (Re complexes) were entered for analysis.

The data set architecture is based on two well-established coordination motifs, fac-[Re­(CO)_3_]^+^, including both mononuclear and dinuclear species. These organometallic compounds feature a variety of mono- and bidentate organic ligands with diverse structural, electronic, and steric properties.

A total of 228 cases were used for the sequential analysis performed. For the ML model development, a subset of 119 complexes with complete descriptors and biological activity data was used. To ensure reliable model evaluation and reduce the risk of overfitting, given the relatively small data set size, we applied a 5-fold cross-validation strategy. The Multi-Layer Perceptron^43^ was employed as the neural network model. MLP is a supervised learning algorithm and one of the simplest types of feed-forward networks, as illustrated in Figure 1. In feed-forward neural networks, the units (or nodes) are arranged in layers without forming sequential loops. This contrasts with recurrent neural networks (RNNs),^44^ where loops allow the network to feed information back into itself. MLP learns a mapping function by training on a data set, determining the relationship between input and output dimensions. Unlike logistic regression, MLP includes one or more nonlinear hidden layers between the input and output layers. These hidden layers enable the network to capture complex patterns in the data.

**Figure 1:**
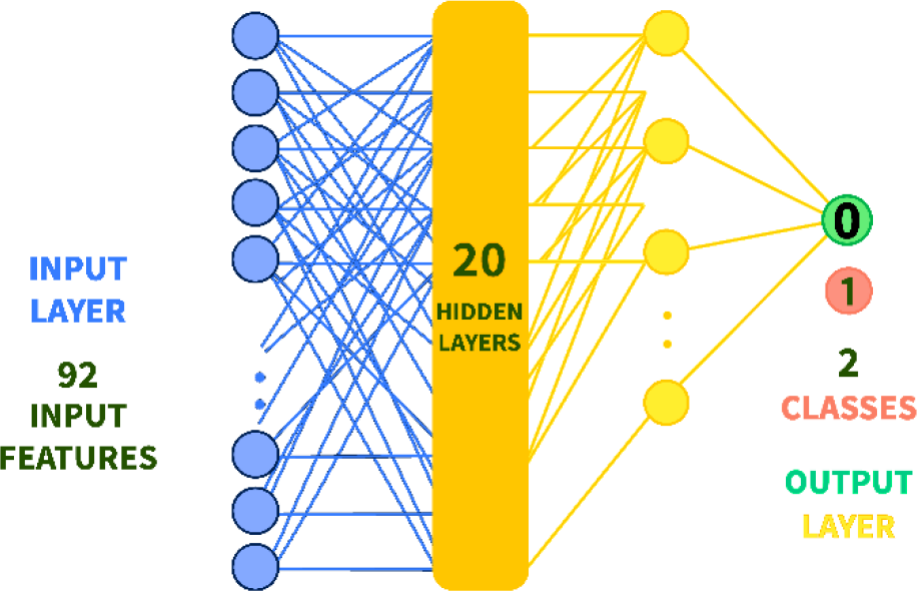
Multilayer perception with two hidden layers. Left picture: In the input layer, input feature values are used for the input units. The output layer has one unit for each value of the network outputs.

Hyperparameter tuning was conducted to determine optimal values for key parameters of the neural network. For instance, the number of hidden layers was set to 20 after experimentation. The model was trained on a data set containing 119 data rows (input objects), 92 feature columns, and 2 output classes derived from percentage inhibition data converted into binary classification. The data set was split into 75% training data (89 rows) and 25% test data (30 rows). The model’s source code, training, and test sets are available at this repository (https://github.com/mici345/MIC-prediction-model).

Using MLP models for small data sets may seem counterintuitive due to the high risk of overfitting associated with the large number of parameters typically found in neural networks. However, several theoretical and practical considerations justify using MLPs even with limited data.^45^ According to Bartlett’s results,^46^ the generalization performance of MLPs is influenced more by the L1 norm of the weights than by the sheer number of weights. This implies that if proper regularization techniques are applied (such as L1/L2 regularization or weight decay), MLPs can generalize well, even with limited training samples. Unlike traditional models, which rely heavily on data volume, neural networks can capture complex nonlinear relationships in small data sets when appropriately constrained.

A Random Forest is a supervised machine learning classifier with multiple decision trees. Each tree uses a unique, independently sampled vector and the input. The final output of the forest is determined by majority selection for classification or averaging for regression tasks. By combining the predictions of multiple randomly generated trees, RF reduces variance, improves accuracy, and provides robust performance. Additionally, RF naturally provides feature importance metrics, making it highly interpretable for understanding which variables contribute most to the prediction.

SHAP is a game-theory-derived approach for interpreting machine learning models. Unlike traditional feature importance methods that provide global rankings^47–49^ SHAP connects optimal confidence allocation from cooperative game theory with local explanations of individual predictions. It assigns a Shapley value to each feature, representing its contribution to a specific prediction by comparing the model’s output with and without that feature. This allows SHAP to offer global interpretability (feature importance across the entire data set) and local interpretability (feature impact on individual predictions).

### Descriptor Generation Space

We initially used 5666 descriptors to build the model, which represent Re compounds. The AlvaDesc software^50^ was used to generate a descriptor space from the 3D structures of each Re complex. The 3D structures were obtained using geometry optimization at the semiempirical PM6 level. These optimized geometries were then used as input for descriptor calculation in the AlvaDesc software. All structures were preprocessed to ensure correct coordination geometries and ligand connectivity before optimization.

The set of used descriptors includes 0D (with no relation to shape, e.g., molecular weight), 1D (e.g., presence of certain active substructures within the molecule), 2D (e.g., molecular graph representations involving bonds between atoms but not bond lengths), and 3D (e.g., distances between specific atomic pairs in the molecule) ones (details in the Supporting Information). The descriptors contain information that could correlate to a given Re complex’s antimicrobial action. The structure of the input matrix for the ML models often leads to a decrease in predictive accuracy. The reduction techniques are typically employed to minimize noise in the data structure, but this process also entails a loss of information. The data sets were reduced with Principal Component Analysis (PCA) to reduce the descriptor space. PCA is an orthogonal linear transformation that transforms the data into a new coordinate system, where the first direction, corresponding to the most significant variance, becomes the new coordinate axis.^46^ Optimal parameter selection within the descriptor space yielded highly converged accuracies for the trained model. Construction of the initial matrix from the explored chemical database is crucial for developing and validating the model based on 26 newly synthesized complexes. The final reduced set consisted of 119 data points (Re complex), 91 features, and 2 output classes for the bacterial strains, resulting from the reduction step. The correlation heat map for the molecular descriptors used for the ML models is presented in the Supporting Information section.

## Experimental Part

### Reagents and Chemicals

All reagents were obtained from standard sources and utilized without any further purification. The compounds Re­(CO)_5_Br and Re­(CO)_5_Cl were purchased from Sigma-Aldrich. For the validation set, complexes **1a**,^51^ **1b**,^52^ **1c**,^53^ **2a**,^51^ **2b**,^54^ **2c**,^55^ **3b**,^56^ **4b**,^57^ **7a** ^51^ and **7b** ^58^ were synthesized according to published procedures. Complexes **5d**, **6d**, **8d**, and **9d** were prepared according to the method described by Cortat et al.^14^ Complexes **3a**, **3c**, **4a**, **4c**, **8a**, and **9a** were prepared with similar procedures. All complexes were synthesized under an inert (Ar) atmosphere.

### Instruments and Analysis

IR spectra were recorded on a Bruker Tensor II with the following parameters: 16 scans for the background and 32 scans for the sample, with a resolution of 4 cm^–1^ in the 4000–600 cm^–1^ region. UV–vis spectra of the complexes were measured on a Jasco V730 spectrophotometer. A Bruker Advance III 400 MHz spectrometer was used to measure the NMR spectra of the complexes. The corresponding 1H chemical shifts were reported relative to the residual solvent protons. A Bruker FTMS 4.7-T Apex II in positive mode was used to perform the mass analyses.

### Synthetic Procedures

Ligands for complexes **2a**–**c** and **7a**–**b** were synthesized using published procedures.^59–62^ Re­(CO)_5_Br and Re­(CO)_5_Cl were used to prepare the complexes in the validation set. Rhenium precursors and ligands were generally reacted in equimolar ratios and refluxed overnight. After the reactions, the products were filtered and washed with the reaction solvent and diethyl ether. The purity of the complexes (Br or Cl species **3a**, **4a**, **8a**, and **9a**) was confirmed as >95%. Compounds **3c** and **4c** were prepared by suspending **3a** or **4a** in MeOH (HPLC grade) with 1 mol equiv of pyridine and AgOTf (1.2 mol equiv) and refluxing in the dark overnight. After the mixture had cooled to room temperature, it was filtered to discard AgBr and dried in a vacuum oven. The compounds were purified by washing with water, followed by centrifugation. Crystals of **4c** suitable for X-ray diffraction analysis were grown by slow evaporation of a dichloromethane solution at room temperature. Spectrochemical characterization of the complexes is in the Supporting Information. Crystallographic data, CCDC number 2296107, have been deposited at the Cambridge Crystallographic Data Centre.

### Antimicrobial Study

The antimicrobial activity of the complexes was assessed against *S. aureus* ATCC 25923 (methicillin-sensitive, MSSA) and *S. aureus* ATCC 43300 (methicillin-resistant, MRSA) strains, following published protocols.^63^ The measured MIC values are expressed in μM (i.e., MIC 4 = 4 μM, MIC 8 = 8 μM), and can be interpreted as the compound inhibited bacterial growth at a concentration of 4 μM, while “MIC 8” corresponds to an MIC of 8 μM, and so on.

## Results and Discussion

### Synthesis of Metal Complexes

A data set of 119 rhenium tricarbonyl complexes was compiled for this work. In addition to the 119 cases, 26 complexes, which had not been previously evaluated for their antibacterial activity, were synthesized and used as the validation set for the model (Figure 2). Complexes shown in Figure 2 were prepared according to well-established procedures employed in the chemistry of this metal core. [Re­(CO)_3_(NN)­X] species (where NN = diimine ligand and X = halide, Br or Cl) were obtained by reacting commercially available Re­(CO)_5_X with one equivalent of NN in refluxing toluene. Pyridine (py) and clotrimazole (ctz) derivatives of the compounds were prepared by reaction of [Re­(CO)_3_(NN)­X] with AgOTf in the presence of py or ctz, followed by precipitation and HPLC purification if required. Characterization of the new compounds is given in the Supporting Information. In addition to standard characterization techniques, the X-ray structure of **4c** was determined (Supporting Information).

**Figure 2:**
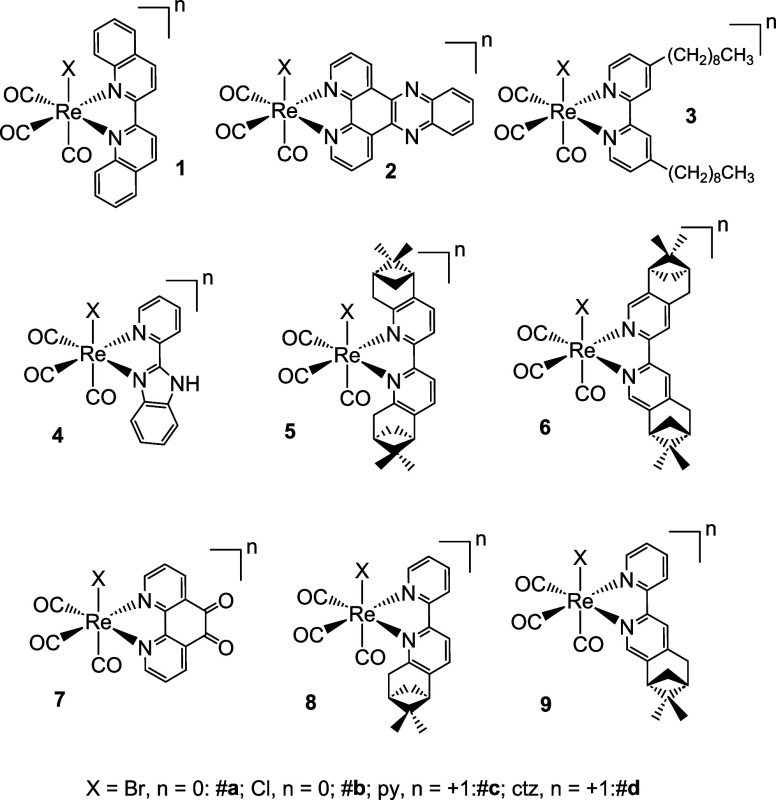
Structures of validation complexes tested for antimicrobial activity against *S. aureus* MSSA and *S. aureus* MRSA strains.

The pipeline for leveraging the prediction model based on antimicrobial data is presented in Figure 3.

**Figure 3:**
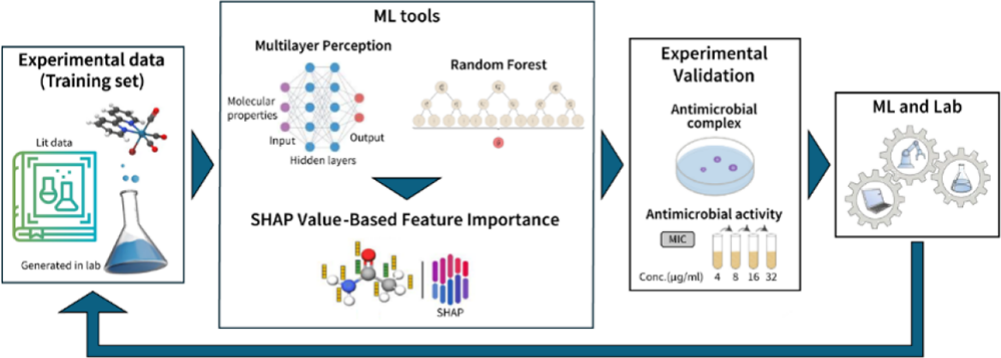
Schematic representation of the workflow.

As a preprocessing step for the classification model presented in Figure S1, the correlation heatmap visually represents the relationships between molecular descriptors used to build the model. Each cell in the heatmap represents the Pearson correlation coefficient between a pair of descriptors, with values ranging from −1 to 1. The diagonal line represents self-correlations (always 1), and the cells are symmetric about this line. The color gradient helps quickly distinguish strong correlations, with red indicating a high positive correlation and blue indicating a high negative correlation. The clustering analysis was performed to understand how descriptors relate to antimicrobial activity according to the MIC values provided, which are supplied in Figure S2 in the Supporting Information section.

Molecular descriptors are mathematical representations of structural or physicochemical properties. Their grouping into clusters reveals patterns that can be directly linked to molecular mechanisms underlying antimicrobial activity or resistance. The primary outcome of the analysis for active and inactive compounds is distinguishing molecular properties posed by the Re complexes. These groups often correspond to or are related to specific mechanisms that affect activity. A hierarchical clustering algorithm grouped descriptors based on similarity (distance metric represented in the dendrogram). Tightly clustered descriptors were selected because they are considered more strongly related and likely to have a significant combined impact on activity. Broader clusters were also considered to capture supporting features with less direct contributions. Looking at the dendrogram (Figure S2a) provided for the active group, the clustering patterns can be analyzed by the cluster groups:

The descriptors in Cluster 1 (distance ∼ 2–5)—SM1_Dz, SpMax, SpMin, VE2_A, VE3_D—are closely grouped, indicating a strong relation within the cluster. The impact of these descriptors on their potential correlation with antimicrobial activity is as follows: The SM1_Dz (Dipole Moment-Based Descriptor) was used for the molecular dipole moment’s contribution, indicating the molecule’s overall charge distribution. The relevance of this fact to antimicrobial activity for a molecule with high dipole moments is often enhanced by electrostatic interactions with bacterial membranes, which are typically negatively charged. The SpMax (Topological Descriptor groups) can be related to molecular shape and maximum branching features, which are correlated with molecular size and complexity, influencing membrane permeability and the ability to disrupt bacterial integrity. Larger or more branched molecules may penetrate lipid bilayers more effectively, enhancing antimicrobial action. A lower SpMin value may correlate with simpler, more compact molecules that exhibit activity, provided other properties, such as polarity or hydrophobicity, are favorable. The VE2_A and VE2_A (Vibrational Energy-Based Descriptors) groups were used to calculate vibrational energy contributions in the molecule, related to atomic masses and bond strengths. Higher vibrational energy indicates increased molecular flexibility, which can enhance interactions with dynamic bacterial environments. The second one incorporates three-dimensional vibrational energy contributions, which is again the effect of the molecule’s ability to adopt and maintain favorable 3D conformations for interacting with bacterial targets. The group of these descriptors can contribute to increased binding efficiency and membrane disruption.

The next cluster is Cluster 2 (Distance ∼ 5–7) of the dendrogram, which contains a group of VSA-related descriptors. These represent the molecular surface area properties related to hydrophobicity, polarity, and interaction with cell membranes. ATS4i is a topological descriptor indicating autocorrelation weighted by ionization potential, associated with the spatial distribution of molecular charge. In Cluster 2, we have an autocorrelation descriptor (ATS4i, GATS6p) that suggests how molecular properties, such as ionization and polarizability, are spatially distributed, which in turn influences molecular reactivity and interactions with bacterial targets. GATS6p is related to atomic polarizability and varies across the molecular structure at a lag distance of 6 bonds. The active complexes should exhibit more potent antimicrobial activity when we have high polarizability values.

For Cluster 3 (middle right branch, Distance ∼ 5–7), the descriptors within that cluster include MATS3i: Moran autocorrelation descriptor at lag 3, weighted by ionization potential, which indicates the spatial distribution of molecular reactivity. It represents the spatial distribution of ionization potential related to reactivity, which, in turn, can influence the compound’s ability to interact with bacterial surfaces. This descriptor provides information about the ionization potential (IP) distribution across the structure, specifically considering interactions between atoms separated by three bonds. This can describe whether regions of the molecule with high or low IP are uniformly distributed, which can influence molecular interactions. The next one, SpMin_A, represents a topological descriptor that captures the minimum structural features of the molecule, which may affect its ability to bind to bacterial targets. Suggests that simpler, more compact molecules might have better binding affinity or target specificity.VE1, which is, again, a vibrational energy descriptor. Indicates how the molecule will be flexible, which could be used to measure the degree of efficiency when interacting with the bacterial membranes.

The last Cluster 4 (Distance ∼ 10–15) includes the following VE2_A, as seen in Cluster 3; it features a vibrational energy descriptor that captures energy contributions from molecular bonds and interactions. We have one more connected with the vibrational energy VE3_D, but this time, considering 3D contributions. We can assume that energy-related descriptors reflect how molecular energy states influence interaction strength with bacterial targets. The last one, i.e., the CATS3D_09_AA’ descriptor, is the atom-pair class 3D pharmacophore descriptor derived from the CATS (Chemically Advanced Template Search) framework. Focusing on the molecule’s hydrogen-bond donor/acceptor atoms (AA), high CATS3D values in active compounds indicate that pharmacophoric features (e.g., hydrogen bond donors/acceptors, hydrophobic regions) are spatially well-organized in 3D space. In the obtained set of active compounds, we observe high CATS3D values, while the reverse trend is observed for nonactive compounds. This analysis can refine structure-based drug design by identifying features that enhance compatibility with bacterial targets in the next stage, such as pocket interactions.

For the second case presented in Figure S2b, where we have nonactive compounds, the distribution of the descriptors is as follows. We are observing the formation of cluster 1 (tightly grouped, distance ∼ 2–5) with the following descriptors: SpMin_A, MATS3i, VE1. SpMin_A belongs to the Burden matrix descriptors, which are related to molecular mass and represent minimal topological features. The second one is MATS3i, which was already observed to capture reactivity; however, here the descriptor values are reversed according to the activity once. The last one is the VE1, reflecting rigid molecular structures. We can then reveal that the following descriptors may correlate with the structures of molecules that are less flexible, less interactive, or less capable of penetrating bacterial membranes, rendering them inactive. Cluster 2 (defined as moderate distance ∼ 5–7) contains VSA-related descriptors and ATS4i. VSA-related descriptors represent surface area properties, which may indicate polar or hydrophobic interactions. The last one, ATS4i, is the topological autocorrelation weighted by ionization potential. The previous cluster 3 (broadly grouped, distance ∼ 10–15) contains descriptors VE2_A and VE3_D, representing the vibrational energy contributions, but might indicate lower adaptability for interaction with bacterial targets and CATS3D_09_AA. We already mentioned the impact of this descriptor. Low values in nonactive compounds suggest that the pharmacophoric features are not optimally positioned in 3D space. The observed poor or limited spatial organization of the features reduces the efficiency of forming strong hydrogen bonding, hydrophobic interactions, or complementary binding.

Based on the hierarchical clustering, active groups emphasize the role of the SpMax, SM1_Dz, and VE2_A descriptors, which are connected to size, flexibility, and electrostatics. Nonactive groups are characterized by SpMin_A (minimal topological features) and MATS3i (limited reactivity).

The box plot depicted in Figure 4 represents the violin plots, which illustrate the distribution of molecular descriptor values based on the hierarchical clustering of active and inactive rhenium complexes.

**Figure 4:**
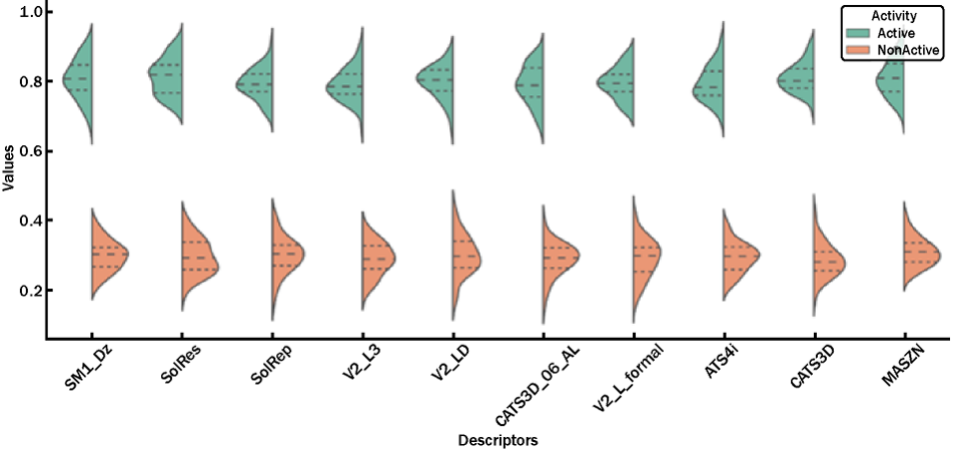
Boxplot representation of selected molecular descriptors stratified by antimicrobial activity.

### Metric for the RF and MLP Assessment Performance

The 119 rhenium tricarbonyl complexes used in this work as a data set are split into class 1 Re complexes, defined as a positive class (active antimicrobial metal complexes), and class 0 metal complexes, defined as the negative class (nonactive antimicrobial metal complexes). The emphasis on accurately identifying class 1 metal complexes is deeply connected to the selection and performance level of the selected ML models used in this study. Ensuring that the chosen ML models can reliably distinguish between class 1 and class 0 Re complexes is critical for achieving the main objectives of this paired approach based on experimental and data-driven research. Misclassification, where a Class 1 complex is assigned to Class 0, could result in the loss of potentially valuable new candidates within the pool of Class 0 complexes. This misplacement means these promising candidates would not advance for further experimental testing and validation, thereby missing opportunities to uncover potent antimicrobial metal complexes. Therefore, it is necessary to ensure the accurate prediction of class 1. To achieve this, the performance of ML models will be evaluated using accuracy, precision, recall, and F1-score, which serve as essential metrics. Each of these metrics provides individual insights into the model’s behavior. While accuracy is the most general measure, capturing the proportion of correct predictions across all cases can be misleading in scenarios with imbalanced data sets, where one class significantly outweighs another. This limitation underscores the importance of complementary metrics, such as precision, recall, and F1-score, particularly in specialized applications like identifying class 1 metal complexes. Recall ensures that as many class 1 complexes as possible are identified, while the F1-score balances this with precision to ensure the results remain sustainable. A model with high recall but poor precision would flood the experimental workflow with false positives, while a model with high accuracy but low recall risks discarding valuable candidates. By prioritizing recall and F1-score, the machine learning framework developed in this work is strategically designed to align with the specific demands of antimicrobial activity research based on the Re complexes.

The evaluation metrics were also conducted on the trained model on a separate test data set, with the outcomes assessed using key metrics derived from the confusion matrix and the classification distribution. The trained model’s performance was evaluated using elements of the confusion matrix and the classification report:

True Positives (TP): Correctly predicted class 1 metal complexes.
True Negatives (TN): Correctly predicted class 0 metal complexes.
False Positives (FP): Incorrectly predicted class 1 metal complexes (actually class 0 – nonactive).
False Negatives (FN): Incorrectly predicted class 0 metal complexes (actually class 1 – active).

### Random Forest Model

Based on the heatmap presented in Figure 5, which shows the accuracy, precision, and recall obtained from the Random Forest model for antimicrobial Re complexes targeting MRSA and MSSA, the following behavior for the two strains is evident. For the MRSA, the obtained accuracy values are relatively consistent, ranging from 0.79 to 0.84 across MIC levels. The case of MIC 4 achieves the highest accuracy (0.84), indicating better overall performance at this threshold for MRSA. The accuracy for MSSA follows a similar trend, with values ranging from 0.78 to 0.83.

**Figure 5:**
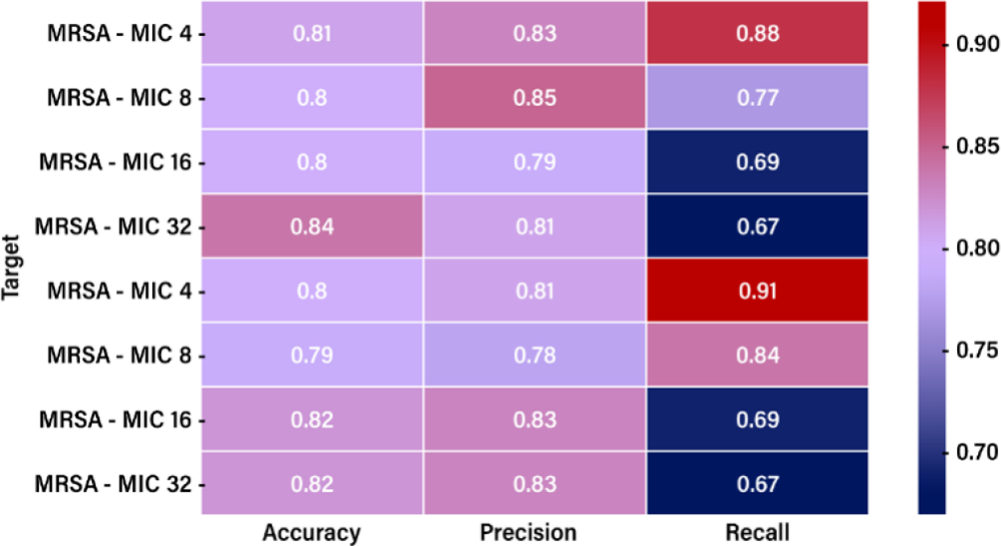
Heatmap for the RF-based accuracy, precision, and recall values for different MRSA and MSSA MIC levels.

The trend evident in the heat map for the Recall metrics is a decrease as the MIC values increase for both strains, MRSA and MSSA. In the case of MRSA, with a maximum MIC of 32, and MSSA, with a maximum MIC of 32, we observe the lowest recall values. Such a pattern in the model can be considered a limitation, as it may cause the model to fail to identify all positive cases when the MIC level increases.

The Precision metrics are generally stable across MIC levels and are often higher than recall, particularly at higher MIC levels. This suggests the models may be more restrained, favoring precision (avoiding false positives) over recall (identifying all true positives).

MSSA – MIC 4 has the most balanced and highest metrics (Accuracy: 0.7983, Precision: 0.8111, Recall: 0.9125), making it the most effective configuration for MSSA.

MRSA – MIC 4 is the best for MRSA, with strong recall (0.8816) and precision (0.8272).

At higher MIC levels (16 and 32), recall drops significantly, particularly for MRSA. This suggests a need for model refinement at these thresholds to capture more true positives without sacrificing precision.

MSSA – MIC 32 shows relatively strong precision (0.8333), but recall (0.6667) is low, suggesting a trade-off where positive cases are missed.

Models perform best at lower MIC levels (4) for MRSA and MSSA, achieving high recall and balanced precision. Lower MIC levels often represent more clear-cut cases of antimicrobial susceptibility, where the drug is highly effective, and the microbial population is consistently inhibited.

### Multilayer Perceptron Model

Using the MLP, we predicted the antimicrobial activity, which was quantified in the MIC values for Re complexes. We trained the MLP architecture on the whole set. The obtained scores were computed based on the validation set and demonstrated how the model performed by comparing its predictions to the experimental validation values. The prediction performance was evaluated using the following parameters: accuracy, precision, and recall. Accuracy estimates the frequency with which the model’s predictions are correct. As such, it is the ratio of the true cases to all the cases, defined as (TP + TN)/(TP + FP + TN + FN) where TP is the number of true positives, FP is the number of false positives, TN is the number of true negatives, FN is the number of false negatives. The set of labels predicted for a sample must match the corresponding labels in the validation set. Precision indicates how often the model correctly predicts a sample to be positive when it is. It is defined as the ratio of the True Positive to the predicted positive cases. The precision is equal to TP/(TP + FP). It is, intuitively, the ability of the classifier not to label as positive a sample that is negative. Recall quantifies the number of positive predictions made from all positive cases in the data set, equal to TP/(TP + FN). The classifier can intuitively find all the positive samples. The obtained scores are shown in Figure 6

**Figure 6:**
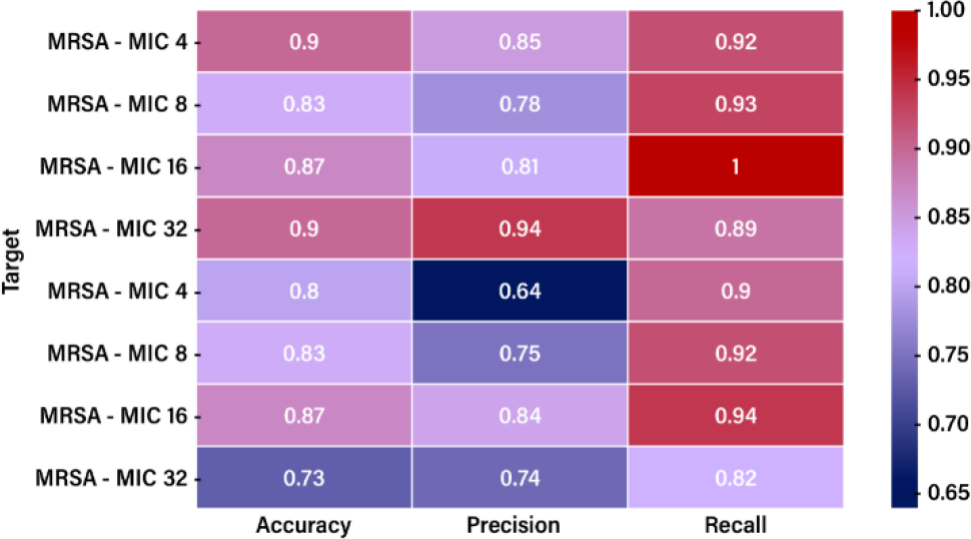
Heatmap for the MLP-based values.

### RF and ML Performance on the Validation Set

A comparison of the two models’ performance on the validation set, presented below (Figure 7a,b), reveals that the MLP model generally outperforms the RF model in terms of recall and overall accuracy. However, the RF model may still be helpful in applications where specificity and precision are more critical.

**Figure 7:**
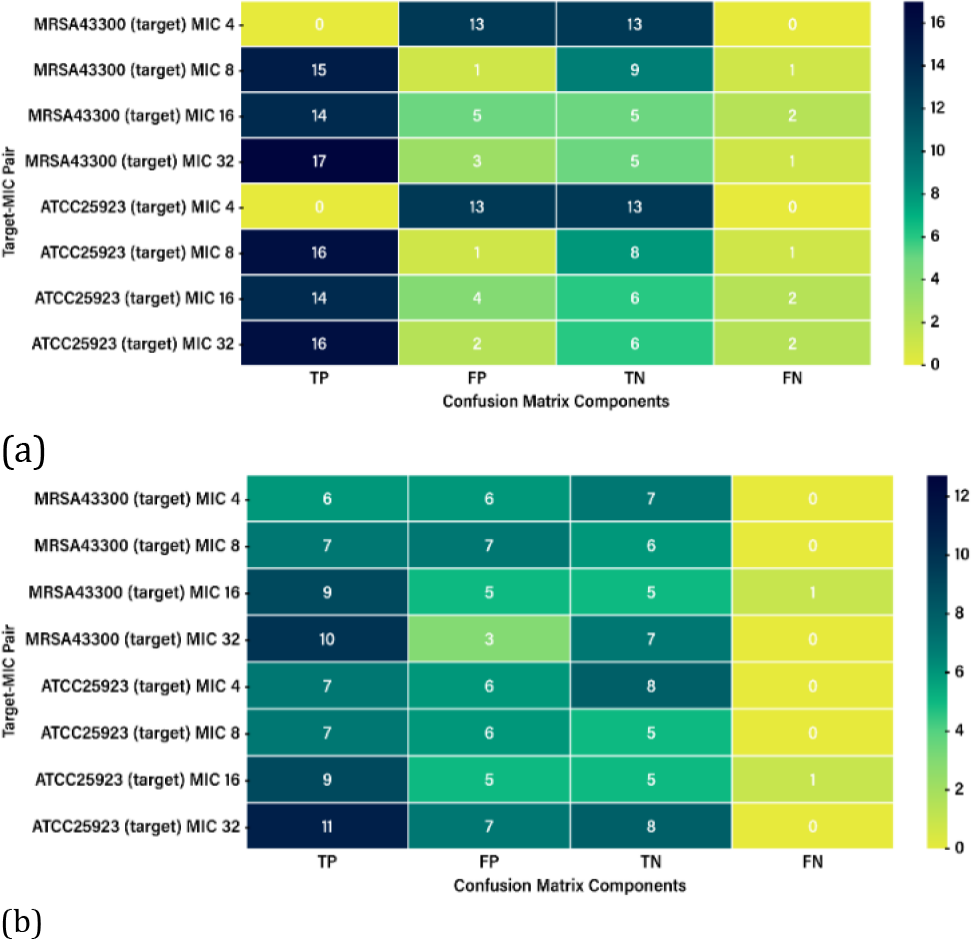
(a) RF performance on the validation set and (b) MLP performance on the validation set.

### Feature Ranking Analysis

The impact of different features and neural network architectures on the prediction quality was systematically evaluated. The distribution of the final importance scores for each feature is shown in Figure S3 in the Supporting Information. Interpreting feature importance in neural networks is inherently challenging. Unlike linear models, neural networks do not offer a straightforward way to trace how input features influence the output, as the relationships are encoded in complex, nonlinear interactions across multiple layers. Specifically, examining the weights alone does not provide clear insight into the role of each feature in the final prediction. To address this, we estimate the importance of the feature presented by the *j* in the following formulation:

$$f_{j}=s-\frac{1}{n}\sum_{i=1}^{n}s_{ij}$$

where *f_j_* represents the importance score for feature *j*, *s* denotes the overall model score, *s_ij_* is the score when feature *j* is perturbed or removed for sample *i*, and *n* is the total number of samples. This approach measures the average change in model performance when feature *j* is excluded or altered, providing an estimate of how critical that feature is for accurate prediction. By permuting, we mean that the values of the feature are randomly permuted between various data rows (molecules). In this way, the importance of a feature is the difference between the baseline score *s* and the average score obtained by permuting the corresponding column of the test set. If the difference is small, then the model is insensitive to permutations of the feature, so its importance is low. On the contrary, the importance of the features is high if the difference is significant. The parameter *n* controls the number of permutations per feature; increasing *n* yields better estimates (we used *n* = 100). The top descriptor with the highest average effect in the prediction model is listed in Table 1. The Kier benzene-likeliness index (BLI) descriptors, which are calculated by dividing the first-order valence connectivity index by the number of non-H bonds (nBO) of the molecule and then normalizing them to the benzene molecule, are proposed to measure the molecule’s aromaticity and were defined as a top-ranked feature of importance. In our approach, the BLI descriptors help explain the antimicrobial activity for the explored complexes by characterizing aromatic moieties that may be critical for interactions with bacterial membranes or intracellular targets. A higher BLI value indicates a greater degree of benzene-like character, which can enhance the lipophilicity and membrane permeability of the complex, allowing it to penetrate bacterial cells more effectively. This suggests that BLI descriptors can serve as important indicators when predicting or rationalizing the antimicrobial potency of Re complexes, especially those designed with aromatic ligands or scaffolds. The BLI descriptor is important, and it is true for all cases except the last case (*S. aureus* MSSA (target) MIC 32).

**Table 1:** Top-Scored Descriptors for Each of the Target Cases

**target**	**categories of descriptors**	**description**
*S. aureus* MRSA MIC 4	BLI - topological indices^64^	The Kier benzene-likeliness index is an aromaticity index calculated from molecular topology.
*S. aureus* MRSA MIC 8	BLI - topological indices^64^	
*S. aureus* MRSA MIC 16	BLI - topological indices^64^	
*S. aureus* MRSA MIC 32	BLI - topological indices^64^	
*S. aureus* MSSA MIC 4	BLI - topological indices^64^	
*S. aureus* MSSA MIC 8	BLI - topological indices^64^	
*S. aureus* MSSA MIC 16	BLI - topological indices^64^	
*S. aureus* MSSA MIC 32	edge adjacency indices^65^	Spectral moment of order 13 from the augmented edge adjacency matrix. Weighted by the resonance integral (structural properties of the graph)

A second method for feature assessment was applied to investigate the effect and robustness of the obtained BLI descriptor, which was ranked as the top. The ranking was performed using Random Forest,^66^ trained on a random forest of 200 classification trees, and stored the out-of-bag information for predictor importance estimation. The critical values are sorted and presented in File S2. Other features of high relevance include edge adjacency indices (e.g., eigenvalue or spectral mean absolute deviation indices, such as Eig05_EA (ri), Eig05_AEA (ed), or SpMAD_EA (dm) and SM13_AEA (ri)). These descriptors are derived from the molecular graph and capture topological and electronic characteristics by quantifying how atoms are connected and how their interactions propagate through the molecular structure. Specifically, they relate to the distribution of atomic properties along molecular edges, reflecting subtle differences in molecular shape, symmetry, and electronic distribution. The relationship between the BLI and topological indices is as follows: while the BLI emphasizes aromaticity-driven interactions, the edge adjacency indices reflect how variations in molecular connectivity and electronic structure can influence the reactivity of the Re complex to interact with biomolecular targets. The strong relevance of both sets of descriptors suggests that a synergistic balance between aromatic character and electronic topology contributes significantly to the antimicrobial efficacy of the Re complex.

### SHAP-Based Feature Impact Evaluation

We applied SHAP analysis to gain a better understanding of the role of individual features, presenting the top 20 descriptors in rankings (Figures S4 and S5 in the Supporting Information). In active compounds, high-impact descriptors such as ChiA_B­(i) and ChiA_B(s)—connectivity indices—suggest that branching and atom-type path configurations are critical for enhancing interaction with bacterial targets. Likewise, descriptors such as those we’ve already commented on, BLI consistently rank high, underscoring the role of aromaticity. Additional high-ranking descriptors like MATS2i, MATS 3p, and SpMin2_Bh­(p) reflect favorable polarizability and charge distribution, which enhance electrostatic interactions with negatively charged bacterial membranes.

In contrast, nonactive compounds tend to show high values for descriptors like Eig05_AEA (ed), IC4, and CIC3, which are associated with graph-based complexity and resonance properties. These features may contribute to molecular rigidity, inefficient pharmacophore alignment, or limited adaptability, thereby reducing the compound’s ability to engage bacterial membranes effectively. Descriptors such as MaxssO and SpMin1_Bh­(p) appearing in nonactive clusters also indicate less dynamic structures, likely impeding efficient target engagement. We can say that the Active complexes possess more flexible, aromatic, polarized, and topologically optimized structures for biological interaction. Nonactive complexes are often rigid, which could hinder the complex’s ability to adapt to biological environments or interact effectively with bacterial membranes.

### MSSA vs MRSA Descriptor-Based Distinction

SHAP-based feature attribution analysis highlights both shared and strain-specific molecular determinants that influence antibacterial activity predictions for MSSA and MRSA. In MSSA, descriptors like SpMin2_Bh­(p) (hydrophobic potential based on Burden eigenvalues) and SM1_Dz­(m) (related to molecular spatial distribution) dominate, suggesting that flexible, diffusible, and hydrophobic structures more readily interact with MSSA’s membrane or cellular targets. The nature of the molecular descriptors found to be important for MSSA-active compounds, such as SpMin2_Bh­(p) and SM1_Dz­(m), and how these compounds likely interact with MSSA bacteria. MSSA can be inhibited by compounds with a more hydrophobic nature that pass through the membrane. They do not need to be ultrarigid or precise—they need to be membrane-friendly.

In MRSA, distinct descriptors such as Eig05_AEA­(ed) and H_D/Dt gain importance. These descriptors highlight resonance complexity, charge localization, and hydrogen bond donor/acceptor topology, underscoring the requirement for rigid, well-organized pharmacophores that can overcome MRSA’s robust defense mechanisms.

A shared descriptor across both strains is presented in Table 2, which is ChiA_B(s), a connectivity index tied to atom-type path branching, underlining the universal role of structural complexity and topology in antimicrobial performance. Based on SHAP descriptors, we can reveal the following insights: MSSA-active compounds benefit from flexibility and hydrophobicity, whereas MRSA-active compounds require electronic accuracy and rigidity, informing future design of Re complexes targeting specific bacterial resistance profiles.

**Table 2:** SHAP-Derived Molecular Descriptors Distinguishing MSSA and MRSA Activity Profiles

descriptor	status	mechanistic role	descriptor type
ChiA_B(s)	shared	atom-type path index (branching)	connectivity
SpMin2_Bh(p)	MSSA-specific	burden eigenvalue (hydrophobic distribution)	hydrophobicity
SM1_Dz(m)	MSSA-specific	eigenvalue from a diagonal matrix (molecular spread)	geometric/topological
Eig05_AEA(ed)	MRSA-specific	adjacency edge eigenvalue (resonance complexity)	electronic/adjacency
H_D/Dt	MRSA-specific	H-bond donor/acceptor spread	hydrogen bonding/electrostatics

## Conclusions

This study establishes a machine learning-driven framework combined with an experimental approach for predicting the antimicrobial activity of rhenium tricarbonyl complexes, revealing the key molecular descriptors that govern their efficacy. The most influential descriptors emphasize the importance of aromaticity, structural complexity, electronic properties, molecular flexibility, and pharmacophoric organization in determining the distinctive mode of activity of active compounds compared to inactive ones.

The strong correlation between higher aromaticity (BLI), electrostatic interactions (MATS3i, ATS4i), and optimized molecular flexibility (VE2_A, VE3_D) with increased antimicrobial potency underscores the mechanistic role of these features in bacterial targeting. The findings demonstrate that active compounds tend to be more structurally complex, electronically interactive, and hydrophobically favorable, enhancing membrane penetration and bacterial disruption. Conversely, nonactive compounds exhibit lower charge distribution, reduced flexibility, and weaker pharmacophore features, limiting their antibacterial potential. This descriptor-driven insight not only enhances model interpretability but also informs the rational design of next-generation metal-based antibiotics, thereby accelerating the discovery of novel candidates against multidrug-resistant pathogens.

## Supplementary Material







## Data Availability

The code used for the model, along with the training and test sets, is provided in this repository: https://github.com/mici345/MIC-prediction-model

## Notes

Collection of the ranked values for the descriptors based on RF (PDF)
Descriptor list of the MIC values (XLSX)
Excel file of the ranking targets (XLSX)
