# Supplementary material for: Machine Learning-Guided Design of Rhenium Tricarbonyl Complexes for Next-Generation Antibiotics
Source: ACS Bio Med Chem Au. 2025 Sep 4;5(5):870–81. doi: 10.1021/acsbiomedchemau.5c00125 (PMC12531868; doi:10.1021/acsbiomedchemau.5c00125)
Supplement: Supplementary file 1 [file bg5c00125_si_001.pdf]

SUPPORTING INFORMATION FOR

Machine learning-guided design of rhenium tricarbonyl complexes for next-generation antibiotics

Miroslava Nedyalkova[1,2], Gozde Demirci[1], Youri Cortat[1], Kevin Schindler[1], Fatlinda Rahmani[1], Justine Horner[1], Mahdi Vasighi[3], Aurelien Crochet[1], Aleksandar Pavic[4], Olimpia Mamula[5], Fabio Zobi[1*], Marco Lattuada[1**]

[1]Department of Chemistry, Fribourg University, Chemin Du Musée 9, 1700 Fribourg, Switzerland
[2] Swiss National Center for Competence in Research (NCCR) Bio-inspired Materials, University of Fribourg, Fribourg, Switzerland

[3] Department of Computer Science and Information Technology, Institute for Advanced Studies in Basic Sciences (IASBS), 45137-66731 Zanjan, Iran
[4]Institute of Molecular Genetics and Genetic Engineering, University of Belgrade, Vojvode Stepe 444a, 11042 Belgrade, Serbia

[5]Haute Ecole d'Inginerie et d'Architecture Fribourg (HEIA), Institute of Chemical Technology, University of Applied Sciences Western Switzerland HES-SO, CH-1700Fribourg

**marco.lattuada@unifr.ch;

*fabio.zobi@unifr.ch

Table of contents

Correlation map for the molecular descriptors used for the ML models

Dendrograms for the descriptor space

Analytic details of **3a**, **3c**, **4a**, **4c**, **8a** and **9a** – page S1-S2

IR spectra (solid state) of **3a**, **3c**, **4a**, **4c**, **8a** and **9a** – Figure S1 – page S2

UV-Vis spectra of **3a**, **3c**, **4a**, **4c**, **8a** and **9a** – Figure S2 – page S3

^{1}H-NMR spectra of **3a**, **3c**, **4a**, **4c**, **8a** and **9a** – Figures S3-S8 – page S3-S6

Antibacterial activity of different Re complexes against *S. aureus* wild-type strain (MSSA, left), and *S. aureus* methicillin-resistant (MRSA) strain – Figure S9 – page S6

Crystallographic details of **4c** – Figures S10 – page S7-S8

Table of the statistical metrics for assessment of the model – Table S4, page S9

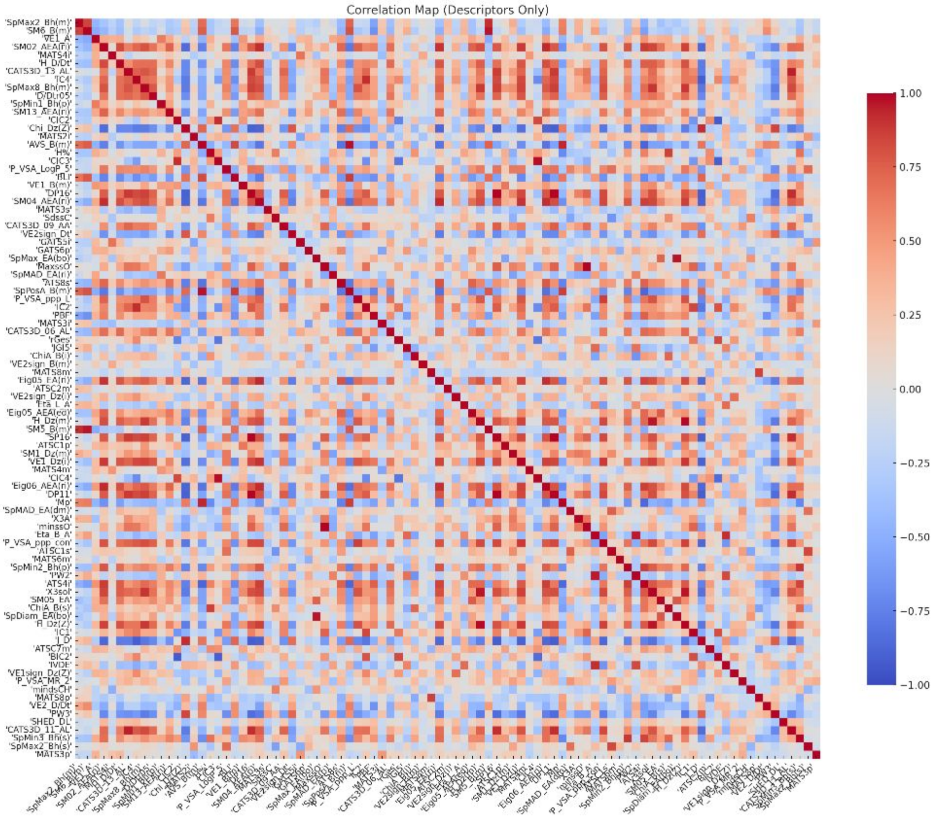

Fig. S1 Correlation heatmap of 91 molecular descriptors.

The correlation heatmap visually represents the relationships between molecular descriptors used to build the model. Each cell in the heatmap represents the Pearson correlation coefficient between a pair of descriptors, with values ranging from -1 to 1.

The diagonal line represents self-correlations (always 1), and the cells are symmetric about this line. The color gradient helps quickly distinguish strong correlations, with red indicating a high positive correlation and blue indicating a high negative correlation. Clustering analysis provides a systematic approach to understanding how descriptors relate to antimicrobial activity, as shown in the MIC values provided in Fig. S1 below.

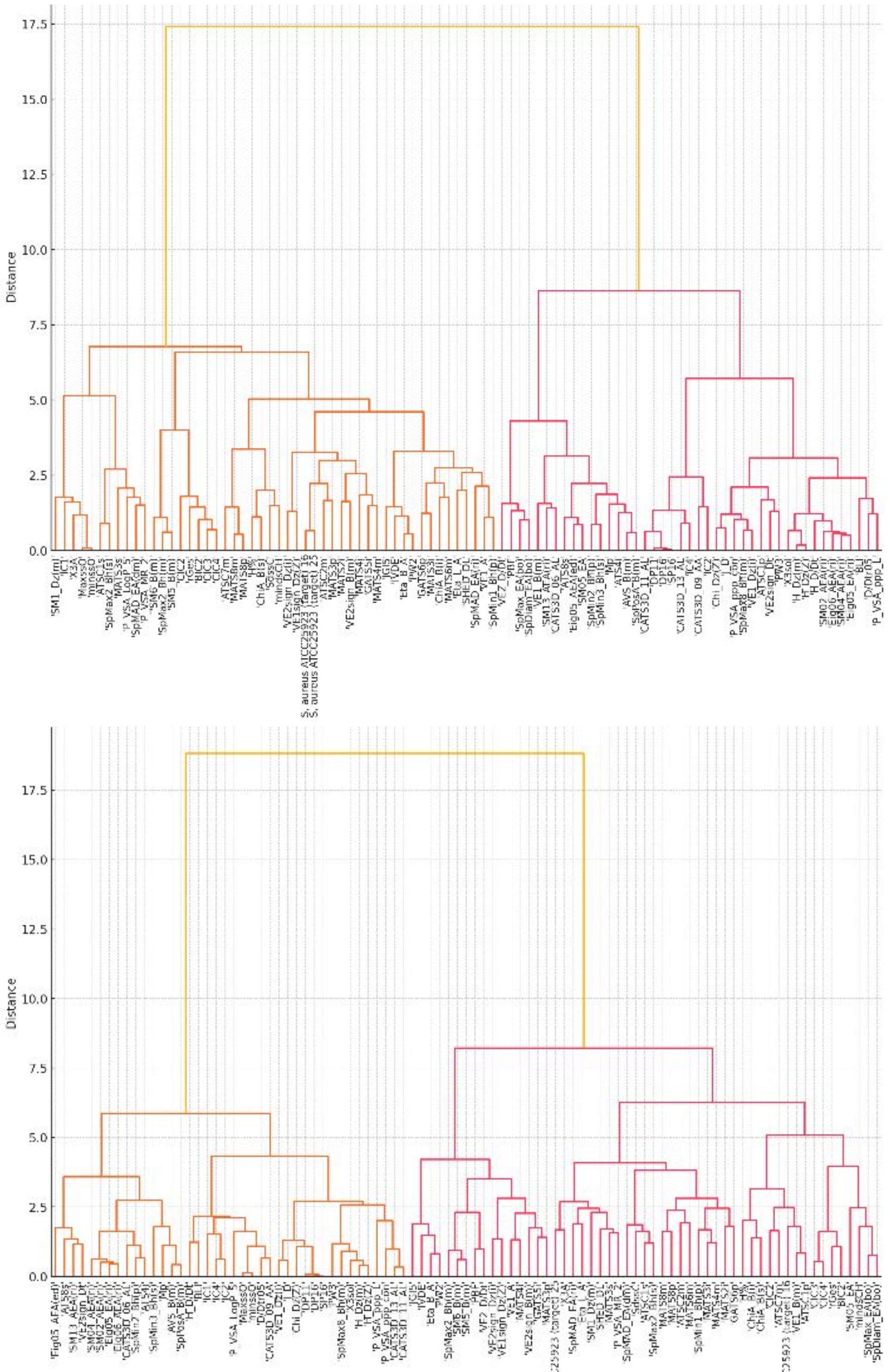

Fig. S2. Hierarchical clustering dendrogram for a) active and b) non-active compounds

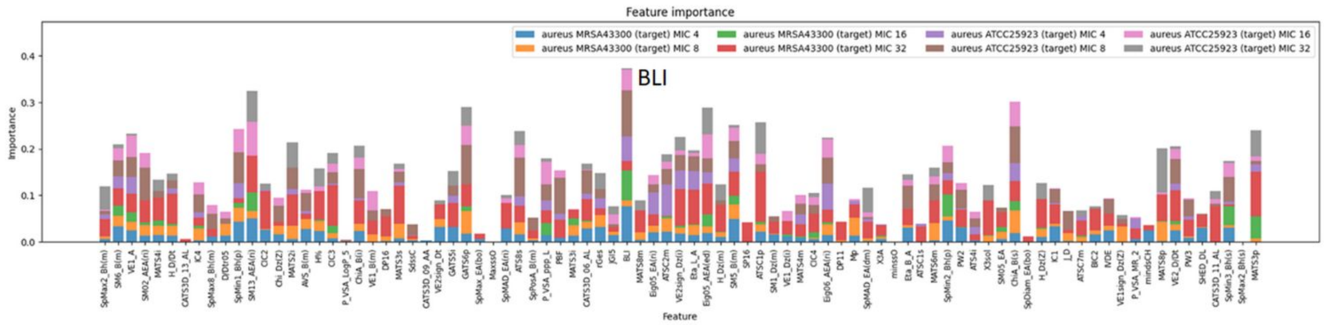

Fig S3. Descriptors ranking

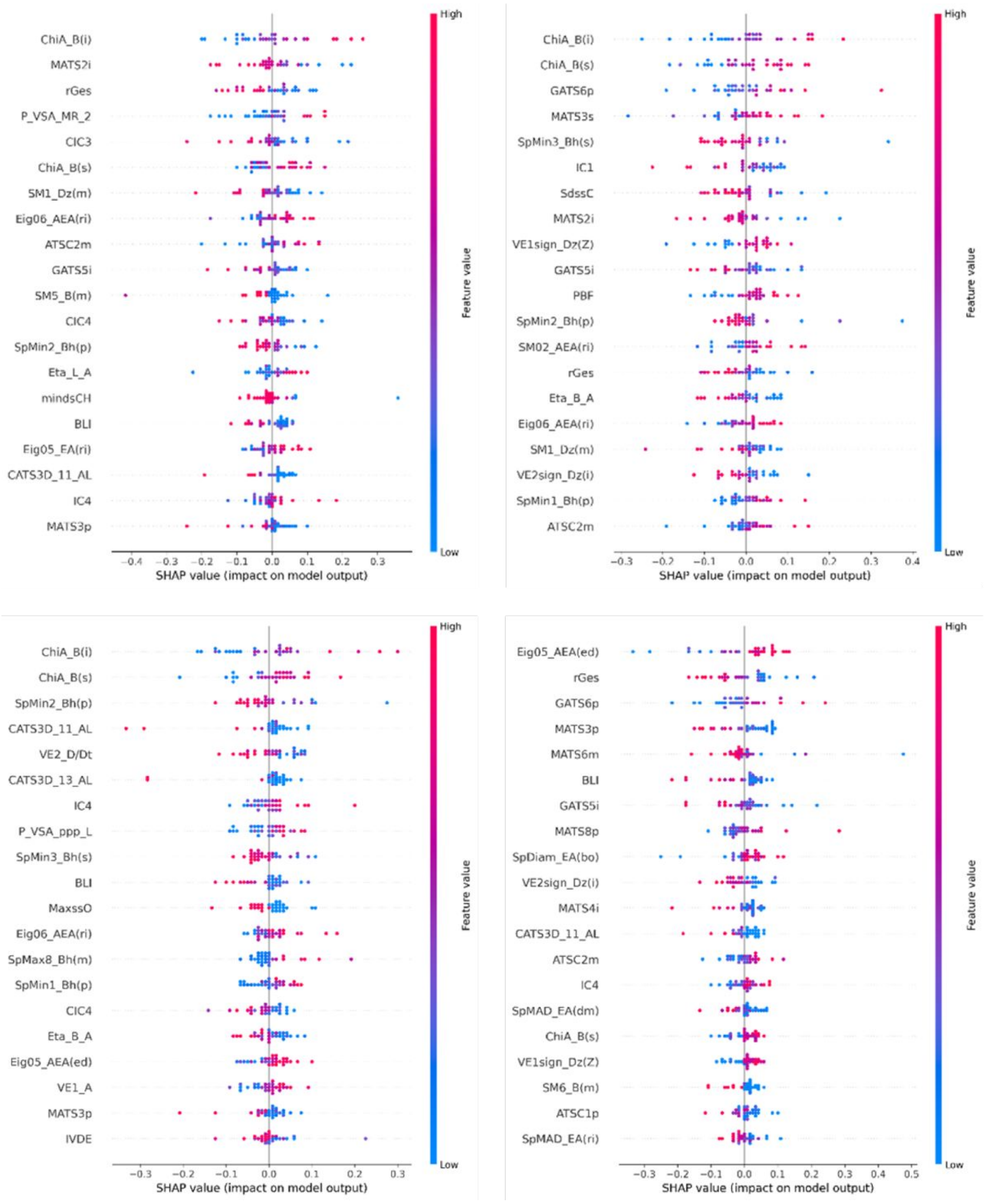

Fig. S4 SHAP summary plot – *S. aureus* MSSA MIC 4,16,32 - top features. Higher feature values (red) often correlate with positive impacts on activity, while lower values (blue) can have the opposite effect.

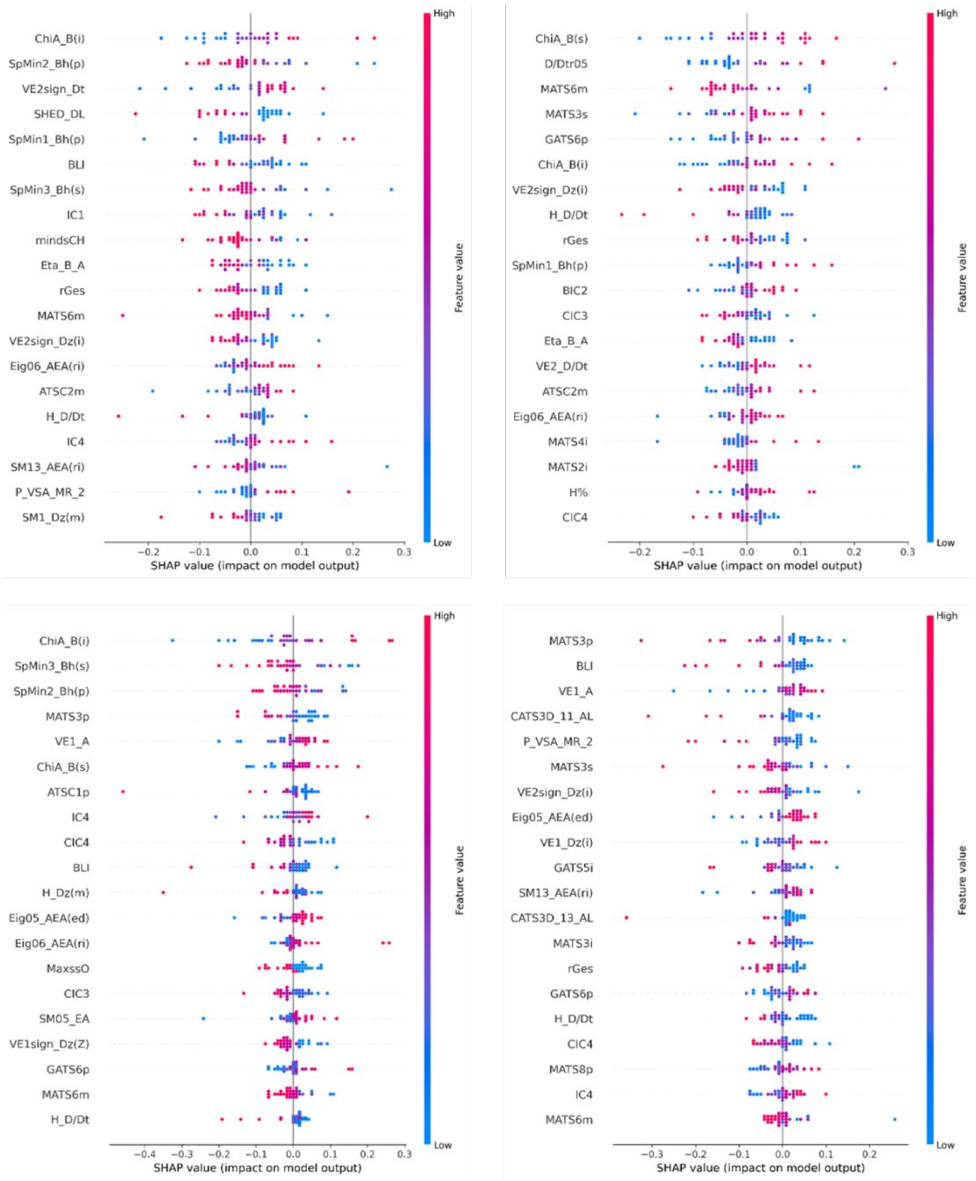

Fig. S5 SHAP summary plot – *S. aureus* MRSA MIC 4,16,32 - top features. Higher feature values (red) often correlate with positive impacts on activity, while lower values (blue) can have the opposite effect.

***fac*-[Re(CO)$_3$(dinonyl)Br] (3a).** 4,4′-Dinonyl-2,2′-bipyridine-Re(CO)$_3$Br complexes. Heptane was used as a reaction solvent. Yellow powder, yield 85.8%. IR (solid, v_{CO}, cm^{-1}): 2015.63, 1909.15, 1888.87. UV-Vis (MeOH, λ_{max} [nm]): 358, 290, 244. ^{1}H NMR (400 MHz, CDCl$_3$): δ = 8.92 (d, J=5.7 Hz, 2 H), 7.96 (d, J=1.1 Hz, 2 H), 7.32 (dd, J=5.6, 1.6 Hz, 2 H), 2.78 - 2.85 (m, 4 H), 1.72 (d, J=7.7 Hz, 4 H), 1.23 - 1.46 (m, 24 H), 0.86 - 0.93 ppm (m, 6 H).

***fac*-[Re(CO)$_3$(dinonyl)Py]$^+$ SO$_3$CF$_3^-$ (3c).** 4,4′-Dinonyl-2,2′-bipyridine-Re(CO)$_3$Py complexes. ??? powder, yield 75.3%. IR (solid, v_{CO}, cm^{-1}): 2028.21, 1905.26. UV-Vis (MeOH, λ_{max} [nm]): 317, 304, 251. ^{1}H NMR (400 MHz, CD$_2$Cl$_2$): δ = 8.97 (d, J=5.7 Hz, 2 H), 8.31 (s, 2 H), 8.17 (d, J=5.0 Hz, 2 H), 7.85 (t, J=7.8 Hz, 1 H), 7.55 (d, J=5.7 Hz, 2 H), 7.31 - 7.37 (m, 2 H), 2.88 (dd, J=8.7, 6.2 Hz, 4 H), 1.69 - 1.78 (m, 4 H), 1.24 - 1.43 (m, 24 H), 0.85 - 0.91 ppm (m, 6 H). ESI-MS (ACN): *m/z*, 758.0 [M]$^+$, measured; calculated for [C$_{36}$H$_{49}$N$_3$O$_3$Re]$^+$: 758.01.

***fac*-[Re(CO)$_3$(PBzIm)Br] (4a).** 2-(2′-Pyridyl)benzimidazole-Re(CO)$_3$Br complexes. Yellow powder, yield 89.5%. IR (solid, ν_{CO}, cm^{-1}): 2020.94, 1889.09. UV-Vis (MeOH, λ_{max} [nm]): 340, 238. ^{1}H NMR (400 MHz, d-DMSO): δ = 14.79 (br. s., 1 H), 9.10 (d, J=5.5 Hz, 1 H), 8.54 (d, J=7.7 Hz, 1 H), 8.37 - 8.45 (m, 1 H), 7.83 - 7.88 (m, 1 H), 7.76 - 7.82 (m, 2 H), 7.54 - 7.61 ppm (m, 2 H).

***fac*-[Re(CO)$_3$(PBzIm)Py]$^+$ SO$_3$CF$_3$$^-$ (4c).** 2-(2′-Pyridyl)benzimidazole-Re(CO)$_3$Py complexes. ?? powder, yield 55%. IR (solid, ν_{CO}, cm^{-1}): 2027.15, 1901.19. UV-Vis (MeOH, λ_{max} [nm]): 342, 230. ^{1}H NMR (400 MHz, CDCl$_3$): δ = 14.48 (br. s., 1 H), 9.14 (d, J=6.1 Hz, 1 H), 9.00 (d, J=7.9 Hz, 1 H), 8.28 (t, J=7.9 Hz, 1 H), 8.16 (s, 2 H), 8.02 (d, J=6.7 Hz, 2 H), 7.80 (t, J=7.8 Hz, 1 H), 7.67 - 7.72 (m, 1 H), 7.59 - 7.64 (m, 2 H), 7.25 ppm (m, 2 H). ESI-MS (ACN): *m/z*, 544.7 [M]$^+$, measured; calculated for [$C_{20}H_{14}N_4O_3Re$]$^+$: 544.56.

***fac*-[Re(CO)$_3$(5,6pin)Br] (8a).** 5,6 pinene bipyridine-Re(CO)$_3$Br complexes. Yellow powder, yield 87.4%. IR (solid, ν_{CO}, cm^{-1}): 2012.01, 1876.28. UV-Vis (MeOH, λ_{max} [nm]): 333, 319, 251. ^{1}H NMR (400 MHz, CD$_3$CN): δ = 9.05 (d, J=5.6 Hz, 1 H), 8.33 (d, J=8.2 Hz, 1 H), 8.11 - 8.19 (m, 2 H), 7.70 (t, J=7.5 Hz, 1 H), 7.54 - 7.60 (m, 1 H), 3.39 - 3.58 (m, 2 H), 2.97 - 3.04 (m, 1 H), 2.72 - 2.82 (m, 1 H), 2.53 (d, J=2.9 Hz, 1 H), 1.45 (d, J=2.9 Hz, 3 H), 1.23 - 1.35 (m, 1 H), 0.64 - 0.70 ppm (m, 3 H).

***fac*-[Re(CO)$_3$(4,5pin)Br] (9a).** 4,5 pinene bipyridine-Re(CO)$_3$Br complexes. Yellow powder, yield 86.9%. IR (solid, ν_{CO}, cm^{-1}): 2016.69, 1913.78, 1885.45. UV-Vis (MeOH, λ_{max} [nm]): 325, 310, 292, 248. ^{1}H NMR (400 MHz, CD$_3$CN): δ = 9.00 (d, J=4.0 Hz, 1 H), 8.55 (s, 1 H), 8.35 (d, J=7.9 Hz, 1 H), 8.23 (s, 1 H), 8.15 (t, J=7.9 Hz, 1 H), 7.55 - 7.60 (m, 1 H), 3.19 (d, J=2.4 Hz, 2 H), 3.05 (s, 1 H), 2.80 (s, 1 H), 2.38 (s, 1 H), 1.45 (s, 3 H), 1.25 (d, J=10.0 Hz, 1 H), 0.68 ppm (d, J=5.1 Hz, 3 H).

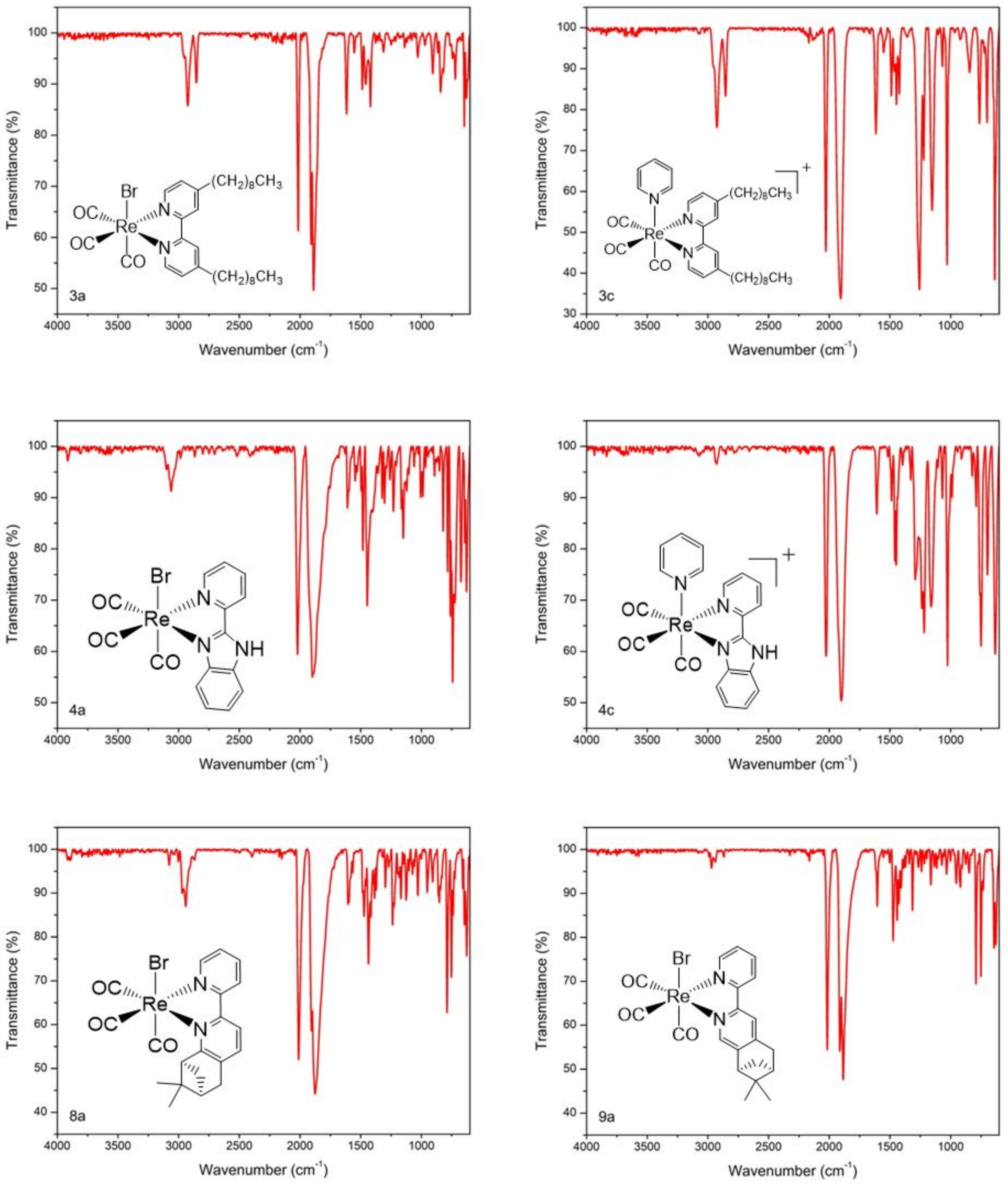

Figure S6. IR spectra (solid state) of **3a, 3c, 4a, 4c, 8a** and **9a** complexes

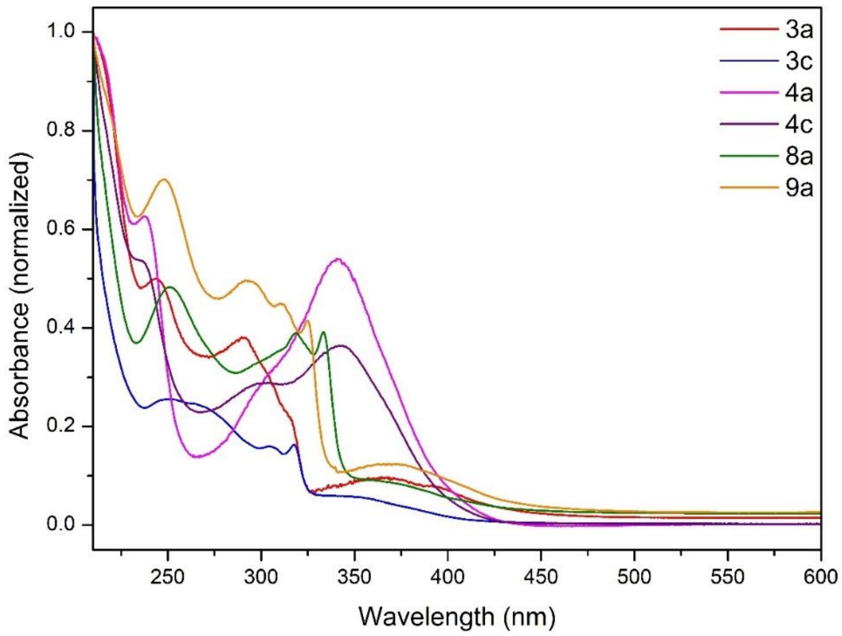

Figure S7. UV-Vis spectra of **3a**, **3c**, **4a**, **4c**, **8a** and **9a** complexes in MeOH

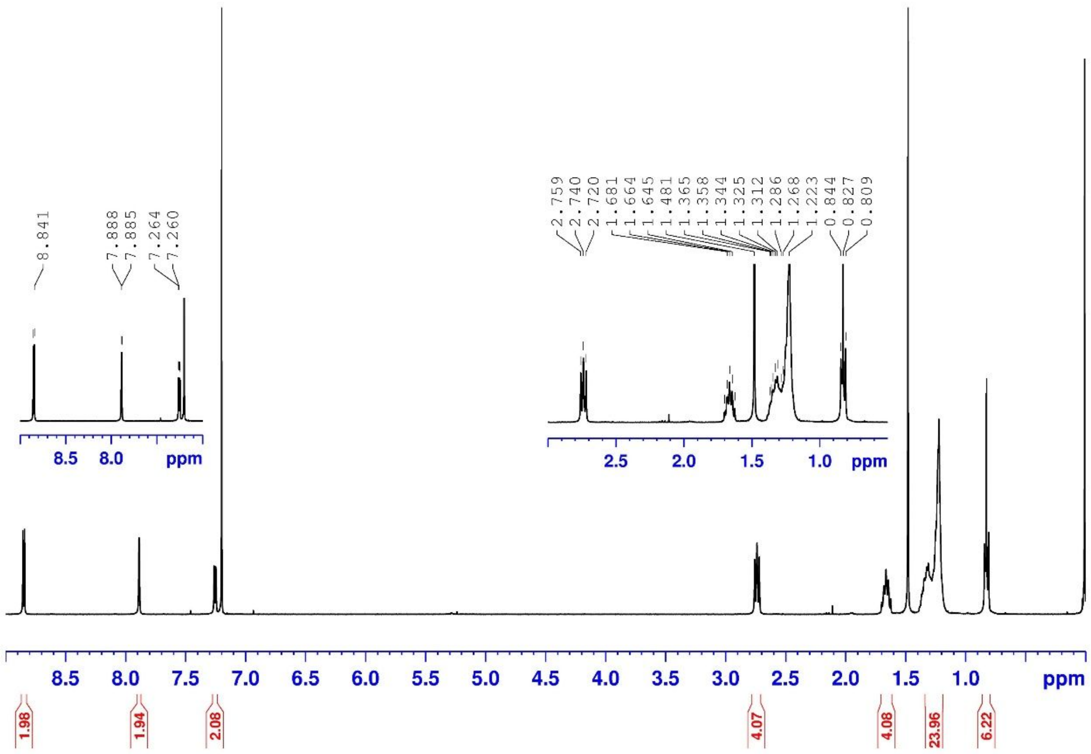

Figure S8. ^{1}H NMR spectrum of **3a** (in $CDCl_3$).

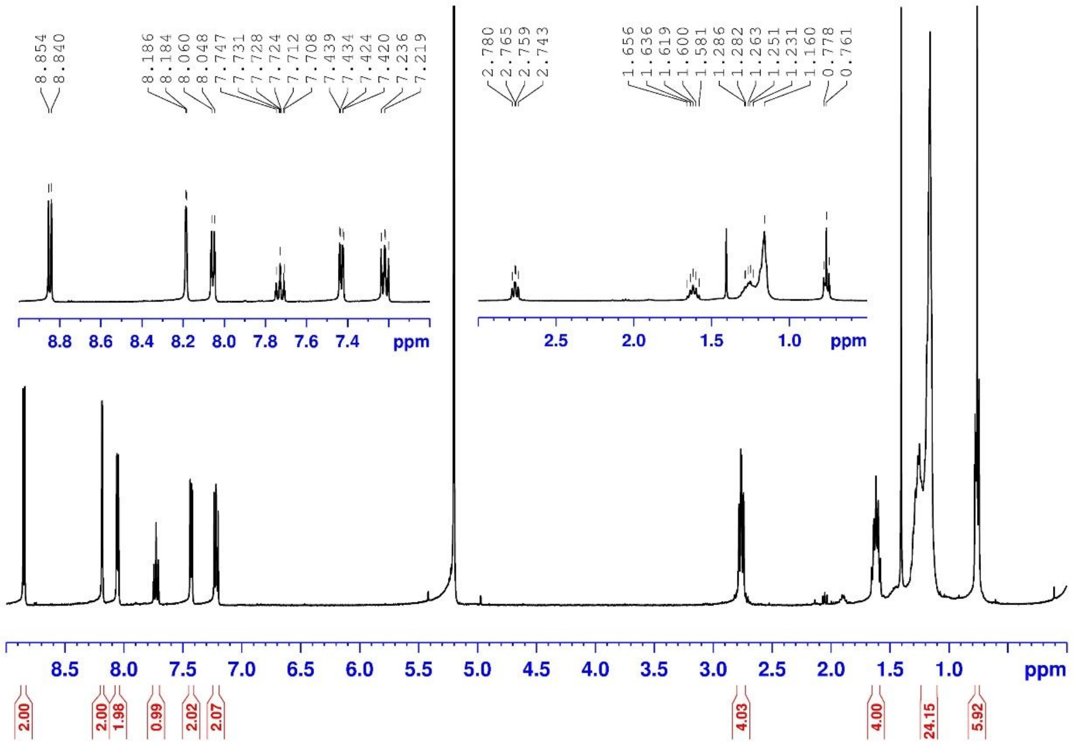

Figure S9. ^{1}H NMR spectrum of **3c** (in CD_2Cl_2).

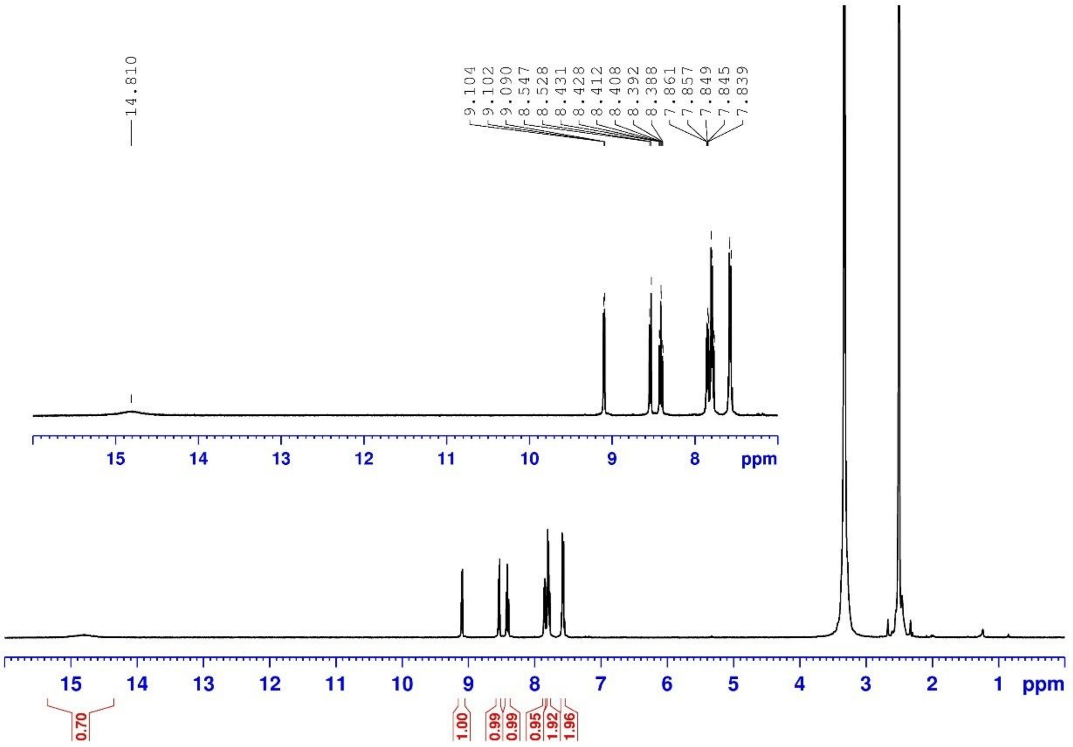

Figure S10. ^{1}H NMR spectrum of **4a** (in d-DMSO).

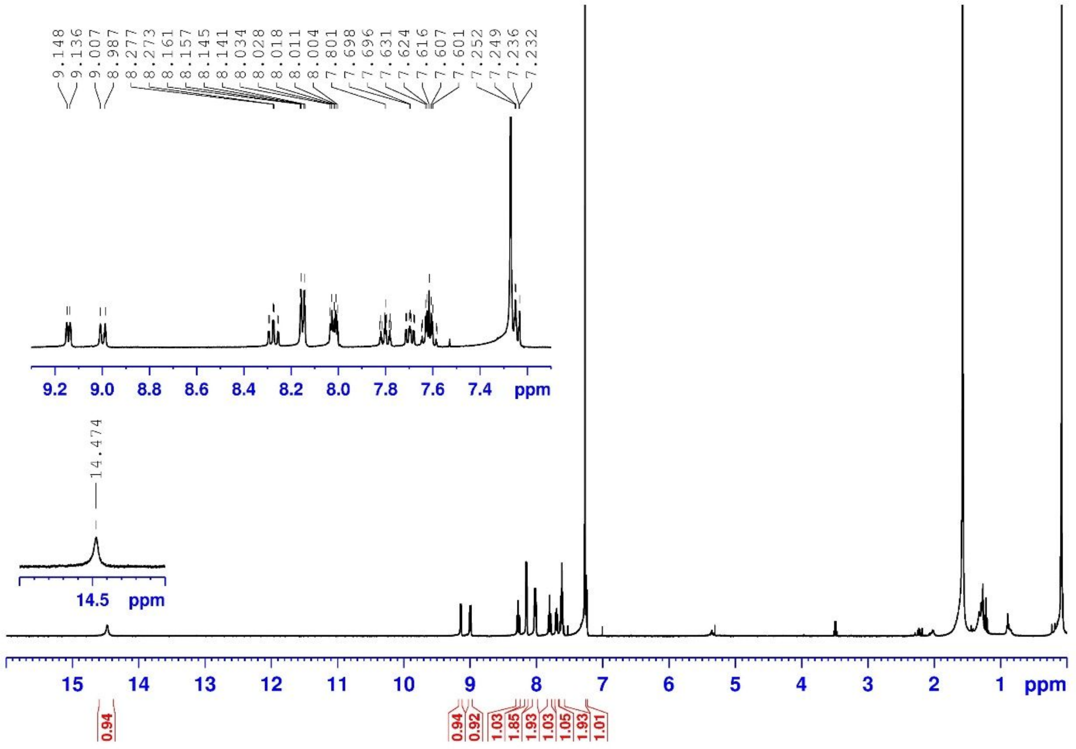

Figure S11. ^{1}H NMR spectrum of **4c** (in $CDCl_3$).

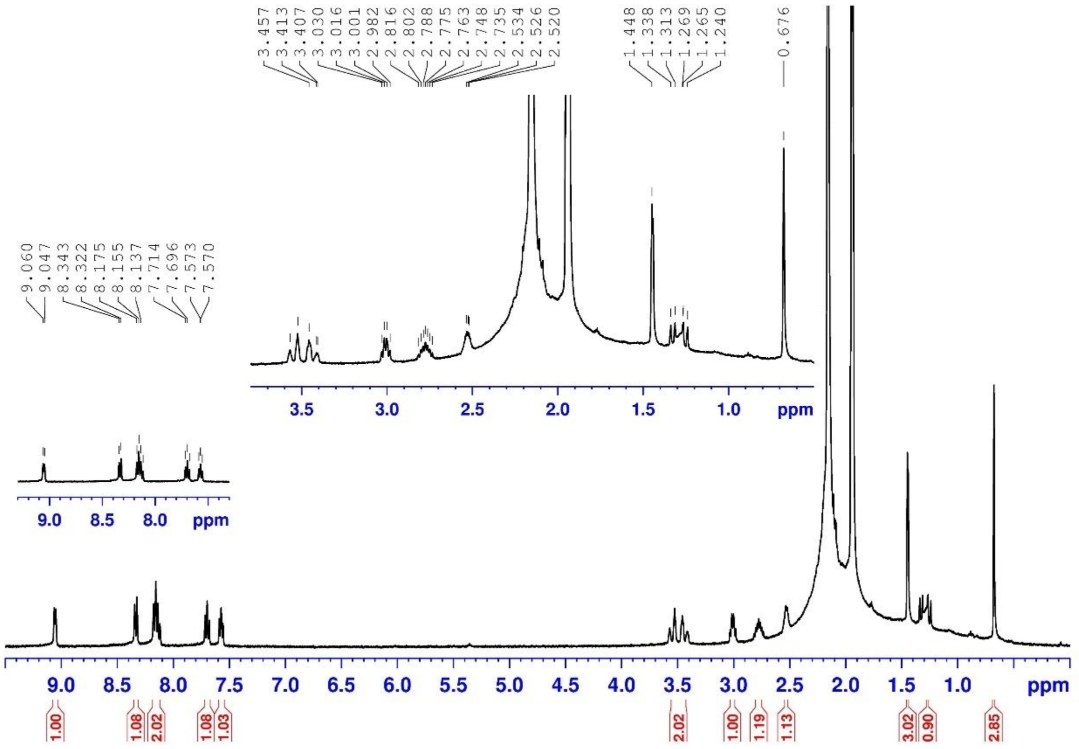

Figure S12. ^{1}H NMR spectrum of **8a** (in CD_3CN).

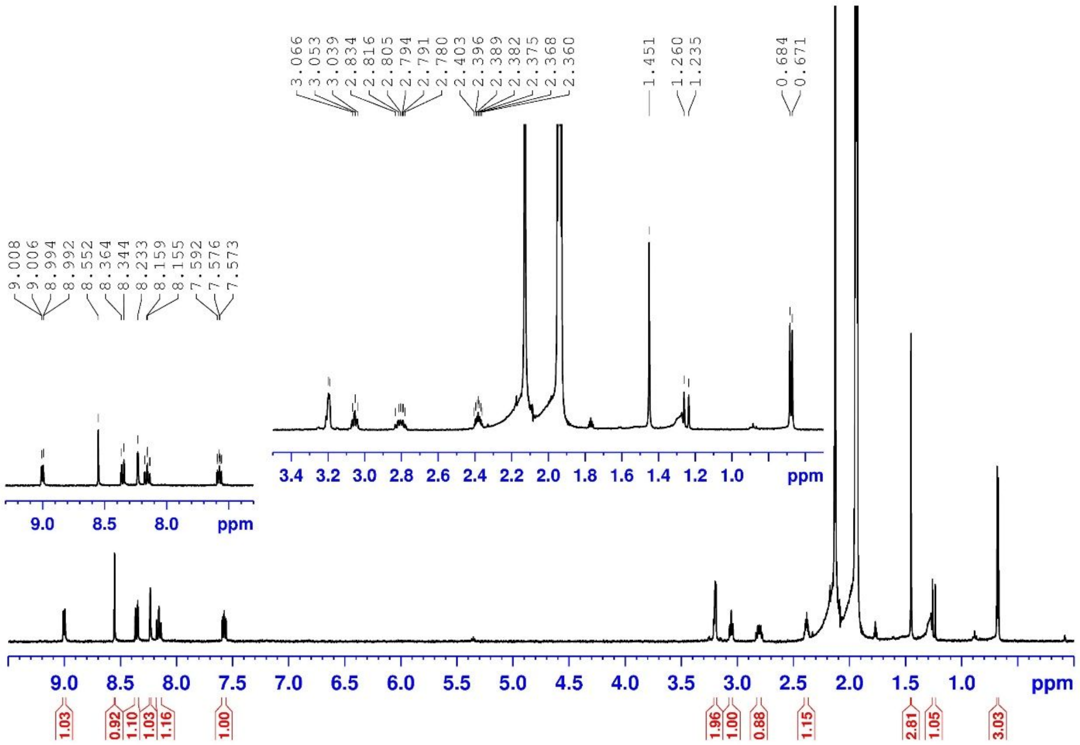

Figure S13. ^1H NMR spectrum of **9a** (in CD_3CN).

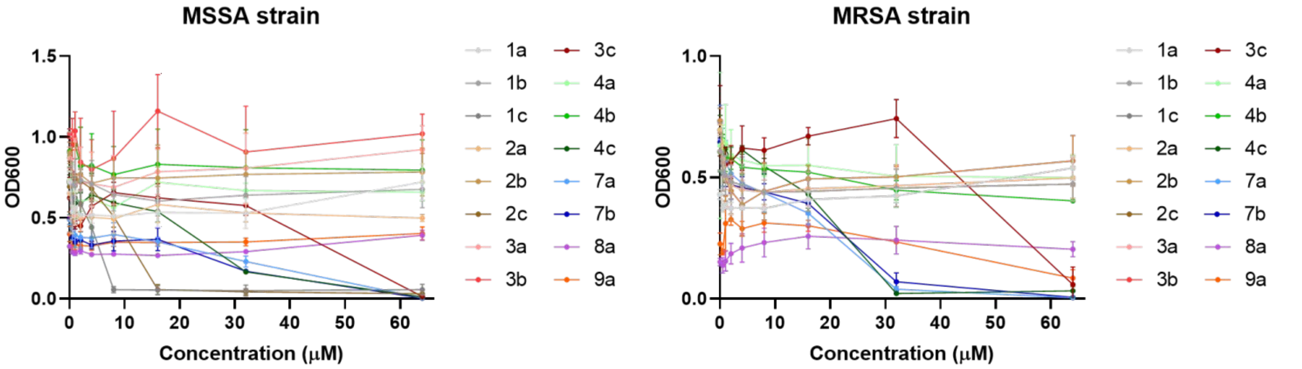

Figure S14. Antibacterial activity of different Re complexes against *S. aureus* wild-type strain (MSSA, left), and *S. aureus* methicillin-resistant (MRSA) strain.

Table S1: Crystal data and structure refinement for 4c.

Identification code	4c
Empirical formula	$C_{21}H_{14}F_3N_4O_6ReS$
Formula weight	693.637
Temperature/K	250(2)
Crystal system	triclinic
Space group	P-1
a/Å	9.5117(4)
b/Å	11.1656(4)
c/Å	11.7463(4)
α/°	91.327(3)
β/°	90.437(3)
γ/°	109.873(3)
Volume/Å^3	1172.74(8)
Z	2
ρ_{calc}g/cm^3	1.964
μ/mm^{-1}	11.610
F(000)	661.9
Crystal size/mm^3	0.16 × 0.123 × 0.07
Radiation	Cu Kα (λ = 1.54186)
2Θ range for data collection/°	8.42 to 139.02
Index ranges	-11 ≤ h ≤ 8, -13 ≤ k ≤ 11, -13 ≤ l ≤ 14
Reflections collected	32432
Independent reflections	4284 [R_{int} = 0.1205, R_{sigma} = 0.0554]
Data/restraints/parameters	4284/0/325
Goodness-of-fit on F^2	1.054
Final R indexes [I>=2σ (I)]	R_1 = 0.0647, wR_2 = 0.1677
Final R indexes [all data]	R_1 = 0.0664, wR_2 = 0.1705
Largest diff. peak/hole / e Å^{-3}	2.93/-2.66

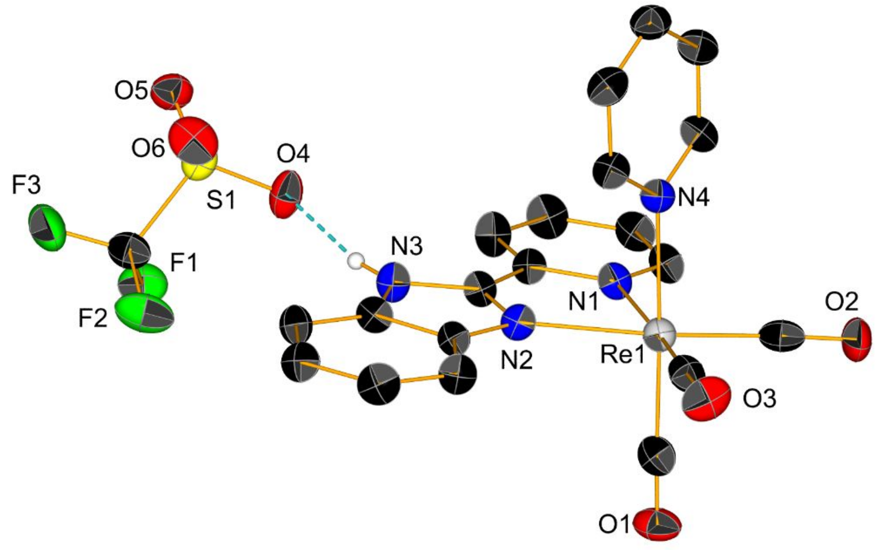

Figure S15. Crystal structures of **4c**. Thermal ellipsoids are at 50% probability. Hydrogen atoms are omitted for clarity.

Table S2 Selected Bond Lengths for 4c.

Atom	Atom	Length/Å
Re1	N1	2.207(7)
Re1	N2	2.184(8)
Re1	N4	2.216(8)
Re1	C18	1.946(13)
Re1	C19	1.907(14)
Re1	C20	1.918(12)
O1	C18	1.120(15)
O2	C19	1.173(16)
O3	C20	1.150(14)

Table S3 Selected Bond Angles for C5-Py.

Atom	Atom	Atom	Angle/˚
N2	Re1	N1	74.4(3)
N4	Re1	N1	83.5(3)
N4	Re1	N2	83.1(3)
C18	Re1	N1	94.7(4)
C18	Re1	N2	94.9(4)
C18	Re1	N4	177.6(4)
C19	Re1	N1	98.4(4)
C19	Re1	N2	172.6(4)
C19	Re1	N4	94.3(4)
C19	Re1	C18	87.5(5)
C20	Re1	N1	173.1(4)
C20	Re1	N2	99.7(4)
C20	Re1	N4	92.3(4)
C20	Re1	C18	89.4(5)
C20	Re1	C19	87.3(5)

Table S16: **Highly correlated descriptor pairs**

The table below lists the highly correlated descriptor pairs, explaining the correlation and potential redundancy of each pair. These descriptors were identified because their correlation coefficients were close to 1, indicating a strong linear relationship.

Descriptor Pair	Correlation Coefficient	Explanation
**SM6_B(m)'**<br>**SM5_B(m)'**	~1.00	These descriptors are highly correlated, suggesting they capture very similar structural information. SM descriptors often represent structural molecular indices that may be related to bond properties, molecular size, or specific electronic features. This high correlation implies redundancy, and one of these descriptors could be removed without significant loss of information.
**SP16'**	~0.99	SP descriptors typically represent shape or surface area properties, while DP

**DP16'**		descriptors might indicate dipole-related properties. The strong correlation suggests that molecular shape consistently influences the dipole moment for these compounds.
**DP11'**<br>**DP16'**	~0.99	Both descriptors are dipole-related. Their high correlation indicates a relationship between the dipole properties of molecules at different conformational states or sizes.
**CATS3D_13_AL'**<br>**CATS3D_11_AL'**	~0.98	Both descriptors are related to 3D molecular alignment. Their correlation suggests that they capture similar spatial information about atomic contributions in the molecular structure.
**VE1_B(m)'**<br>**VE1_A'**	~0.97	These descriptors represent volumetric or spatial properties. Their high correlation indicates that they provide overlapping information regarding the size and shape of molecules.

The highly correlated descriptor pairs indicate potential redundancy in the dataset. Removing one descriptor from each highly correlated pair could reduce dimensionality and improve model performance without sacrificing predictive accuracy. Specifically:

- **SM6_B(m)'** or **SM5_B(m)'** could be removed.
- **SP16'** or **DP16'** could be removed,
- **DP11'** or **DP16'** could be removed,
- **CATS3D_13_AL'** or **CATS3D_11_AL'** could be removed.
- **VE1_B(m)'** or **VE1_A'** could be removed,

Table S5. Statistical metrics for the model assessment

TARGET	TP	FP	TN	FN	Specificity	Accuracy	PPV	Recall	MCC	F Score	Total number	Sensitivity
MRSA43300 (target) MIC 4	6	7	9	0	0.563	0.682	0.462	1.00	0.510	0.632	22	1
MRSA43300 (target) MIC 8	7	8	7	0	0.467	0.636	0.467	1.00	0.467	0.636	22	1
MRSA43300 (target) MIC 16	9	7	5	1	0.417	0.636	0.563	0.90	0.354	0.692	22	0.9
MRSA43300 (target) MIC 32	10	5	7	0	0.583	0.773	0.667	1.00	0.624	0.800	22	1
ATCC25923 (target) MIC 4	7	6	9	0	0.600	0.727	0.538	1.00	0.568	0.700	22	1

ATCC25923 (target) MIC 8	9	8	5	0	0.385	0.636	0.529	1.00	0.451	0.692	22	1
ATCC25923 (target) MIC 16	9	7	5	1	0.417	0.636	0.563	0.90	0.354	0.692	22	0.9
ATCC25923 (target) MIC 32	13	9	0	0	0.000	0.591	0.591	1.00	0	0.743	22	1

Accuracy: (TP + TN) / (TP + FP + TN + FN)
Specificity: TN/(TN+FP)
Precision: TP/(TP + FP)
Recall: TP/(TP + FN)
MCC: (TP × TN – FP × FN)/sqrt((TP + FN)(TP + FP)(TN + FP)(TN + FN))
F1: 2 × ((precision × recall)/(precision + recall))
Sensitivity: TP/(TP+FN)
